# Universal hidden order in amorphous cellular geometries

**DOI:** 10.1038/s41467-019-08360-5

**Published:** 2019-02-18

**Authors:** Michael A. Klatt, Jakov Lovrić, Duyu Chen, Sebastian C. Kapfer, Fabian M. Schaller, Philipp W. A. Schönhöfer, Bruce S. Gardiner, Ana-Sunčana Smith, Gerd E. Schröder-Turk, Salvatore Torquato

**Affiliations:** 1 Institute of Stochastics, Karlsruhe Institute of Technology (KIT), Englerstr. 2, 76131 Karlsruhe, Germany; 2 Department of Physics, Princeton University, Princeton, NJ 08544 USA; 3 Division of Physical Chemistry, Ruđer Bošković Institute, Bijenička 54, 10 000 Zagreb, Croatia; 4 School of Engineering and Information Technology, Murdoch University, 90 South St, Murdoch, WA 6150 Australia; 5 PULS Group, Department of Physics and Interdisciplinary Center for Nanostructured Films, Friedrich-Alexander-Universität Erlangen-Nürnberg, Cauerstraße 3, 91058 Erlangen, Germany; 6 Department of Chemistry, Princeton University, Princeton, NJ 08544 USA; 7 Institut für Theoretische Physik I, Friedrich-Alexander-Universität Erlangen-Nürnberg, Staudtstr. 7, 91058 Erlangen, Germany; 8 School of Computer Science and Software Engineering, The University of Western Australia, 35 Stirling Highway, Crawley, WA 6009 Australia; 9 Department of Applied Mathematics, Research School of Physical Sciences and Engineering, The Australian National University, Canberra, ACT 0200 Australia; 10 Department of Chemistry, Department of Physics, Princeton Institute for the Science and Technology of Materials, and Program in Applied and Computational Mathematics, Princeton University, Princeton, NJ 08544 USA

## Abstract

Partitioning space into cells with certain extreme geometrical properties is a central problem in many fields of science and technology. Here we investigate the Quantizer problem, defined as the optimisation of the moment of inertia of Voronoi cells, i.e., similarly-sized ‘sphere-like’ polyhedra that tile space are preferred. We employ Lloyd’s centroidal Voronoi diagram algorithm to solve this problem and find that it converges to disordered states associated with deep local minima. These states are universal in the sense that their structure factors are characterised by a complete independence of a wide class of initial conditions they evolved from. They moreover exhibit an anomalous suppression of long-wavelength density fluctuations and quickly become effectively hyperuniform. Our findings warrant the search for novel amorphous hyperuniform phases and cellular materials with unique physical properties.

## Abstract (web summary)

Disordered hyperuniformity implies a hidden order on length scales that can be found in various amorphous materials. Klatt et al. analyse the evolution of random point patterns using Llyod’s algorithm and show that they converge to an effectively hyperuniform state regardless of the initial conditions.

## Introduction

Hyperuniformity^1^ is a geometric concept to probabilistically characterise the structure of ordered and disordered materials. It is defined as the anomalous suppression of density fluctuations on large length scales. For point patterns this is reflected in the vanishing structure factor *S*(*k*) for small wavenumbers *k*, see reference^1^. A point pattern is hyperuniform if *S*(*k*), which is directly observable in *X*-ray, light, or neutron scattering experiments, decays to zero as *k* = 2*π*/*λ* tends to zero (i.e., in the infinite-wavelength limit *λ* → ∞). In the context of amorphous structures, hyperuniformity implies a hidden form of order, in which the system remains macroscopically uniform, despite not being crystalline. The concept of hyperuniformity sheds light on a variety of seemingly unrelated fields, including density fluctuations in the early universe^2^, biological tissue^3^, statistical physics^4–6^, colloidal^7,8^ or granular^9^ packings, microfluids^10,11^, driven nonequilibrium systems^12,13^, photonic band gap materials^14–16^, enhanced pinning in superconductors^17^ and terahertz quantum lasing^18^.

As hyperuniformity is defined as an asymptotic limit $[{\mathrm{lim}}_{k \to 0}S(k) = 0]$, it is hard to establish via computer simulations strict hyperuniformity for a given system. The development of a strict statistical test for hyperuniformity in simulated systems is a hard mathematical problem of great importance, and no such test is currently available. In the absence of a statistical test of hyperuniformity, a standard method for asserting ‘effective hyperuniformity’ (that is, hyperuniformity for all practical intents and purposes) is by numerical evaluation of a hyperuniformity index *H*, defined below. Recent discoveries demonstrate that ‘effectively hyperuniform’ systems (as quantified by the hyperuniformity index *H*) are of interest in their own right both for applications and in theory even if they are not perfectly hyperuniform [*S*(0) = 0] or if their perfect hyperuniformity cannot be strictly established. Such systems are essentially hyperuniform for all practical intents and physically relevant purposes. Examples include glass-forming polymer melts^19^, self-assemblies of polymer-grafted nanoparticles^20^, hyperuniformity in image analysis for experimental data^21^, novel states of amorphous silicon^22,23^, certain phase transitions in amorphous ices^24^ and packings approaching the ideal maximally random jammed state^25^, see Supplementary Table 1 for *H* values.

Point patterns induce partitions of space into cells if to each point a neighbourhood is assigned such that these neighbourhoods, i.e., cells, do not overlap but tile the space. The density fluctuations in the point process obviously affect the size fluctuations of the cells. Cellular partitions appear ubiquitously in science, from physics to biology, and in technology, from telecommunications to urban planning^26^. Identification of cellular partitions of space that extremize certain geometrical properties is a basic problem of fundamental importance. Excellent examples include the Kelvin minimal-cell-interface-area problem^27^ and Kepler sphere-packing problem^28,29^.

Roughly speaking, in the ‘Quantizer problem’^30^, a partitioning of space is sought with similarly-sized ‘sphere-like Voronoi polyhedra’ whose centroids are as close as possible to the Voronoi-cell generating points. More precisely, for a given set of *n* points **z**_1_,…**z**_*n*_ the ‘Voronoi Quantizer’ is the partition of space into cells, where each Voronoi cell *C*_*i*_ consists of all points in space that are closer to **z**_*i*_ than to any other point **z**_*j*≠*i*_. It assigns to a ‘test point’ at **x**, which is chosen randomly in the system, the nearest point **z**_*i*_ in the set of points; put differently, if the input **x** is in **C**_*i*_, the output is **z**_*i*_. The ‘Quantizer energy’ *E*(*C*_*i*_,**z**_*i*_) of a cell *C*_*i*_ and its generating point **z**_*i*_ is then defined as the mean squared error of the quantization for test points that fall inside that cell:

1 $$E(C_i,{\mathbf{z}}_i): = \mathop {\int}\limits_{C_i} ||{\mathbf{x}} - {\mathbf{z}}_i||^2\,d{\mathbf{x}},$$

that is, the cell energy is the second moment of the mass distribution (moment of inertia). In the context of soft materials, this energy functional is reminiscent of the entropic chain stretching free energy of copolymeric self-assemblies in the strong segregation limit^31^. The Quantizer problem is the minimisation of the ‘total energy’, defined as the sum $\mathop {\sum}\nolimits_i E(C_i,{\mathbf{z}}_i)$ of all single-cell contributions^30^.

The energy functional favours tessellations with similarly-sized cells and spherical cell shapes that are centred on the generating points. However, the space-filling nature of the Voronoi partition prevents the cells from adopting the optimal spherical shape. The configuration of points located on the vertices of the body-centred cubic lattice (BCC) is the best known global optimum of this functional^32,33^.

The total energy defines an energy landscape within the space of generating point coordinates. Local minima are necessarily ‘centroidal Voronoi tessellations’ (CVTs) where the centroids (centres of mass) of each cell coincide with the corresponding generating points^34^. CVTs are widely used, with applications including telecommunication, biology, image processing, computer science, and electrical engineering^34–37^. In an earlier study, Gabrielli et al.^38^ showed that while assigning a fixed number of points to idealised periodic and quasiperiodic tiles of the same volume always results into a hyperuniform point pattern, positioning the points in the centroids of the cells results in a particularly strong suppression of large-scale density fluctuations. Whether an amorphous CVT with cell volume fluctuations sufficiently suppresses the density fluctuations to even become approximately hyperuniform is highly non-trivial and addressed in the present work.

Here we demonstrate the emergence of universal effectively hyperuniform amorphous states associated with deep local energy minima in the ‘Quantizer problem’. In particular, these states are characterised by an anomalous suppression of long-wavelength density fluctuations, and are universal with respect to a variety of statistics such as two-point statistics and an associated order metric *τ*, cell energy distributions and Minkowski structure metrics. Note that a comprehensive study of the inherent structures, which are the local energy minima in the energy landscape, in terms of the low-*k* limit of *S*(*k*) is lacking in the literature, even for commonly used potentials such as the Lennard-Jones potential. Moreover, for generic potentials, the expectation is that the properties of the metastable local energy minima depend on the nature of the structure of the initial conditions. There is no a priori reason why one would expect these inherent structures to exhibit a suppression of density fluctuations on large length scales consistent with perfect hyperuniformity.

## Results

### Generation of centroidal Voronoi tessellations

**Fig. 1:**
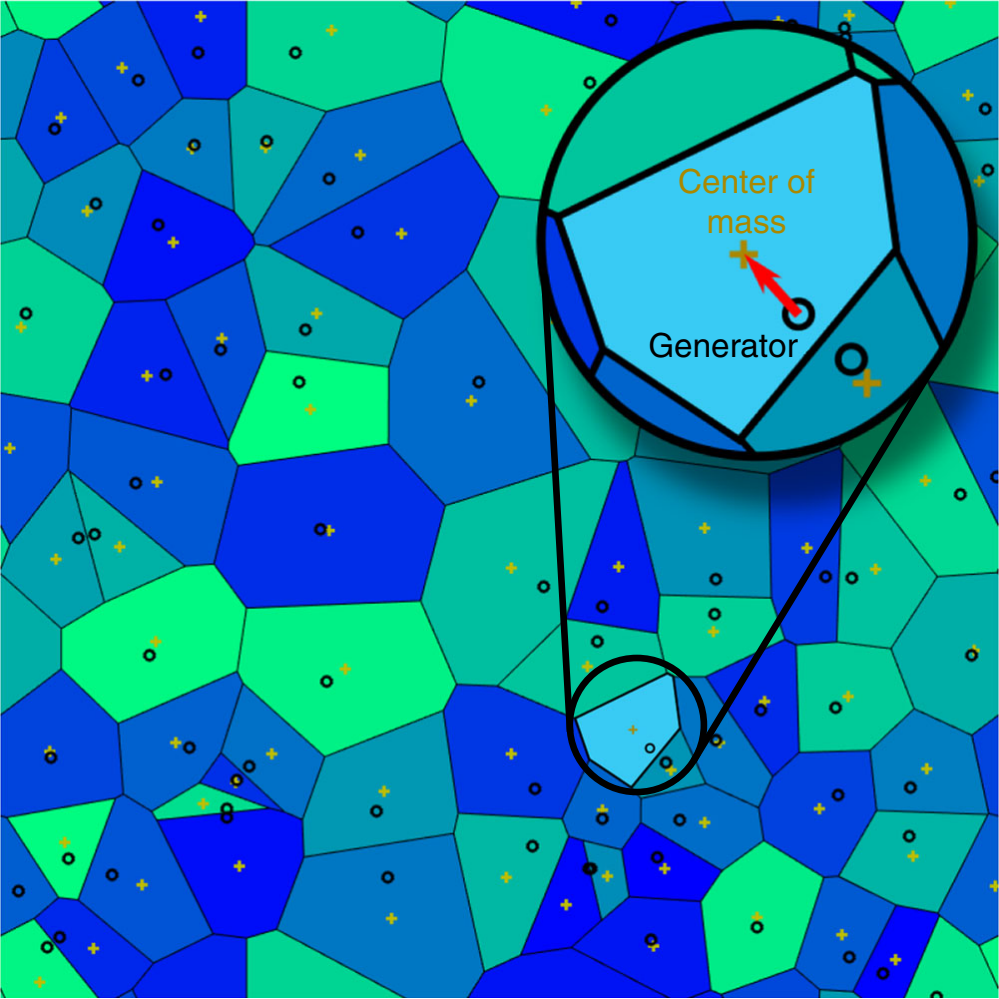
Schematic representation of a Lloyd iteration. Lloyd iterations convert an initial point set to a point set with a centroidal Voronoi diagram. In each iteration, the algorithm first computes the Voronoi cells for all points. Then each point (black circle) is replaced by the centre of mass (yellow cross) of its Voronoi cell

An efficient way to dynamically generate a CVT is via Lloyd iterations^39^ which evolve a given initial set of points by iteratively replacing each point with the centre of mass of its Voronoi cell^40^, see Fig. 1. This corresponds to a gradient descent algorithm^34^, a standard energy minimisation procedure that in general converges to a random minimum in the potential energy surface when starting from different initial conditions.

**Fig. 2:**
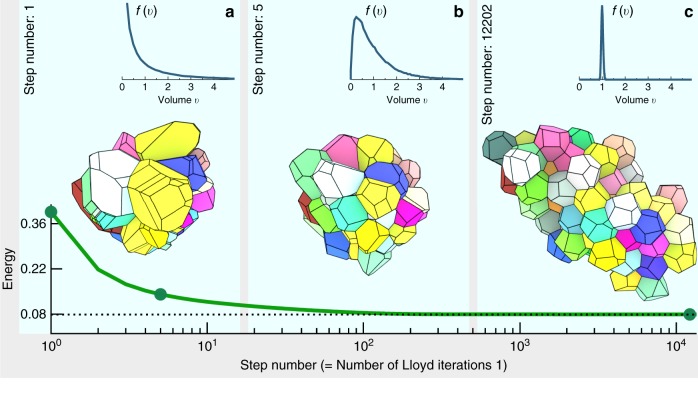
Convergence of Lloyd’s algorithm in 3D. Shown is a subset of a 3D system at step number *N* = 1 (initial tessellation), *N* = 5, and *N* = 12202 (final tessellation); see also the Supplementary Movie 1. The initial configuration is a 3D hyperfluctuating point set. The distributions *f*(*v*) of cell volumes *v* demonstrate the high degree of uniformity in cell volumes in the final states. The dimensionless total energy converges to a value 〈*e*_*t*_〉 ≈ 1.008 × *e*_BCC_ slightly above the value *e*_BCC_ = 0.07854 of the optimal BCC lattice

Here we analyse the evolution of random point patterns under Lloyd iterations, see Fig. 2 and the Supplementary Movie 1, which we carry out for a very broad spectrum of disordered geometries^41^ as initial configurations (see Methods section and Supplementary Fig. 1). They include repulsive or clustering point patterns, isotropic or anisotropic processes, and systems with vanishing or arbitrarily large density fluctuations, from stealthy hyperuniformity (where *S*(*k*) = 0 below a finite threshold *k* < *k*_0_)^6^ to the opposite, hyperfluctuating (or anti-hyperuniform) point configurations (where *S*(*k*) → ∞ for *k* → 0)^42^ that are derived from a hyperplanes intersection process^41,43^.

### Universal effectively hyperuniform amorphous states

**Fig. 3:**
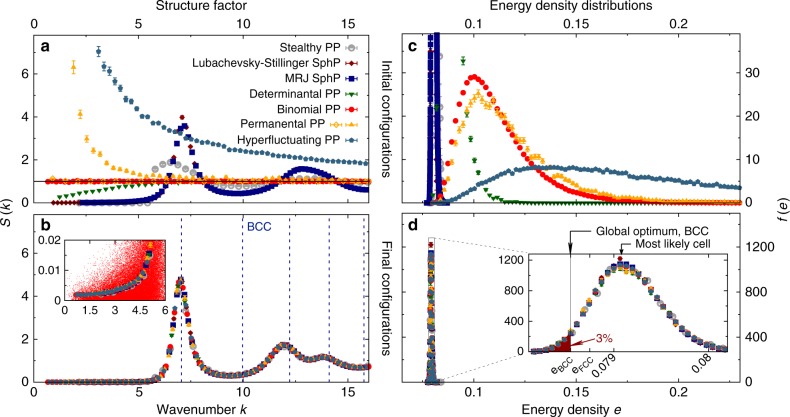
Evidence supporting the universal effectively hyperuniform final states in 3D. The structure factor *S*(*k*) is shown before (**a**) and after (**b**) application of Lloyd iterations. Different symbols represent different initial stochastic models, including anisotropic and hyperfluctuating systems, and including point processes (PP) and sphere packings (SphP), see Methods section. For the final configurations, *S*(*k*) collapses within statistical errors and indicates effective hyperuniformity, in the inset the point cloud (red) shows the unbinned scattering intensity for all allowed wavenumbers for the largest sample (Binomial PP); for log-log plots, see Supplementary Figs. 8 and 9. Frequency distributions *f*(*e*) of cell energy densities *e* (see Methods section) are shown before (**c**) and after (**d**) application of Lloyd iterations; for a semi-log plot, see Supplementary Fig. 6. The distributions of energies after Lloyd iterations have mean 〈*e*〉 ≈ 1.008 × *e*_*BCC*_ and FWHM 0.01 × *e*_*BCC*_. In the final universal configurations, there is a significant proportion (≈ 3%) of cells with a lower energy density than the crystalline optimal BCC structure. The error bars represent the standard error of the mean

Here, we find a striking, unexpected universality for the complex interactions of the Quantizer energy in a detailed structure analysis of its local minima. Figure 3 shows the key result of this paper, the existence of universal effectively hyperuniform and fully amorphous states that are the stable converged solutions of Lloyd’s algorithm in 3D. Even though the initial structure factors differ vastly for the initial structures (Fig. 3a), the final configurations are characterised by a universal form of the structure factor *S*(*k*), within statistical errors (Fig. 3b), and universal two-point statistics (Supplementary Fig. 2). The universality is further confirmed by the *τ* order metric^6^, an integral over *S*(*k*) that measures correlations on all length scales (see Methods section). While for the initial configurations *τ* varies from zero to infinity, it adopts the same value 31 ± 1 for all final configurations (see Supplementary Table 2). As long as there are no crystalline patches in the initial configuration (see Methods section and Supplementary Note 4), all systems studied here converge to the same state within statistical errors.

Even ﻿when the initial configuration is hyperfluctuating, where the structure factor diverges for small wavenumbers, the system quickly becomes under Lloyd iterations effectively hyperuniform (almost stealthy) for all system sizes studied here. The structure factor drops within the first few hundred iterations by up to four orders of magnitude. In the final configurations, after 10^4^ iterations, the structure factor effectively vanishes for small wavenumbers (*S*(*k*) < 3 × 10^−3^ for *k*<3 at unit number density), and the index *H* of effective hyperuniformity, see Eq. (10) in the Methods Section, is among the lowest of all known effectively hyperuniform systems, see Supplementary Table 1. Whether a precise CVT is strictly hyperuniform remains an open question for further research (for details see the Methods section and Supplementary Note 8). Here we demonstrate the strong suppression of density fluctuations on large but finite length scales that is consistent with effective hyperuniformity.

It remains to ascertain that the converged configurations are indeed amorphous across all length scales without any trace of local crystalline structure. We provide three pieces of evidence for the absence of crystallites: (a) the structure factor exhibits no (Bragg) peaks, see Fig. 3b; (b) the absence of local crystalline configurations is confirmed by the distributional properties of a comprehensive set of Minkowski structure metrics^44,45^, see Supplementary Fig. 3 and Supplementary Note 5; and (c) the distribution of cell energies exhibit no over-expression of values corresponding to crystalline motifs, see Fig. 3d.

Despite the optimal structures being crystalline, Lloyd’s algorithm does not converge to these ordered configurations, nor does it converge to partially ordered configurations. Rather, the evolution reaches the universal effectively hyperuniform states described above as its stable converged solution, regardless of the nature of the disordered initial configuration.

An indication why the amorphous universal states, rather than the lower-energy BCC lattice, are the final stable states of Lloyd’s algorithm, is afforded by two considerations: first high energy barriers between basins of nearly degenerate crystalline states and deep local minima (with total energies 〈*e*_*t*_〉 ≈ 1.008 × *e*_BCC_ close to that of the ground state *e*_BCC_, see Methods section) and second the ‘geometric frustration’ of local configurations with energies lower than that of the global ground state, see Methods section.

Both the universality and these two aspects support our conjecture that the final effectively hyperuniform amorphous states represent an infinite set of nearly-degenerate stable local optima for the Quantizer problem, characterised by the same universal energy distributions and two-point statistics. In contrast to a rapid freezing of initial fluctuations, the iterative local optimisation of Lloyd’s algorithm results in large-scale global rearrangements, that suppress density fluctuations on large length-scales. Instead of getting trapped in a multitude of metastable states with a broad distribution of energies, the same intrinsically disordered states appear to emerge as a solution of the optimised Quantizer energy for all of the widely different, amorphous initial conditions.

### Planar tessellations

**Fig. 4:**
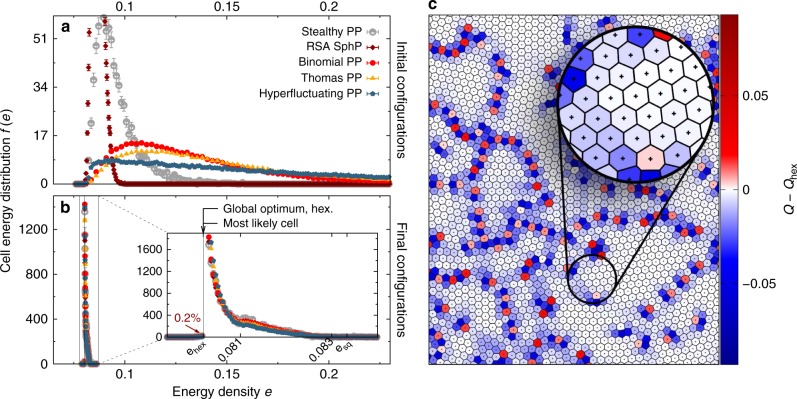
Effectively hyperuniform and universal final configurations in 2D with local crystallites. Frequency distribution *f*(*e*) of cell energy densities *e* (see Methods section) are shown before (**a**) and after (**b**) application of 10^4^ iterations of Lloyd’s algorithm (see Methods section for initial point processes). The error bars represent the standard error of the mean. In the final configurations, which are effectively universal, only a small portion of cells (0.2%) has an energy density lower than that of the (globally optimal) regular hexagon. Here *e*_sq_ is the energy density of the square lattice. **c** Voronoi cells in the final CVT: The colour code indicates the difference between the isoperimetric ratio *Q* = 4*πA*/*P*^2^ of each cell, where *A* is the area and *P* the perimeter of the cell, and the isoperimetric ratio *Q*_hex_ = 0.9069 of the regular hexagon. The local crystallites and their globally amorphous arrangement are clearly visible. For the structure factor of the initial and final configurations, see Supplementary Fig. 4

The nature of the 3D problem is highlighted by juxtaposition to the 2D system. What first meets the eye in 2D is the formation of small ordered domains of nearly regular hexagonal Voronoi cells (Fig. 4c). This dimensionality effect is reflected also in the distribution of cell energy densities (Fig. 4b): The most prevalent cell type in 2D is the optimal hexagonal cell, in contrast to 3D where the optimal BCC cell is not preferred relative to amorphous cell shapes. It is explained first by the fewer degrees of freedom of point configurations in 2D (compared to 3D) and secondly by the behaviour of cells with energy densities lower than the crystalline optimal cell. The fraction of these cells (≈ 0.2%) is not only an order of magnitude smaller than in 3D, but they are also exclusively larger than the average cell (again in contrast to 3D); see Supplementary Fig. 5b.

A subtler, but more surprising observation is the degree to which the 2D configurations remain amorphous compared to typical polycrystals, such as those generated by the Lubachevsky-Stillinger (LS) algorithm. The CVTs exhibit a far faster decay of peak intensities in *S*(*k*), so that the *τ* order metric converges (see Methods section and Supplementary Fig. 7). In contrast to the LS system, the CVT does not enforce a strict minimal distance requirement between adjacent generating points. This leads to lower degrees of order in the crystalline regions (in contrast to the exact regular hexagons of the LS polycrystal) and prevents the formation of point defects. Therefore the final configurations are effectively hyperuniform, again in contrast to the LS system (see Supplementary Figs. 4, 7 and Supplementary Note 8, and the Methods section). Further, the final states are effectively universal for a remarkable range of initial patterns, see Methods section. Actually, in terms of effective hyperuniformity, global amorphousness and universality, the 2D system is remarkably similar to the 3D system, with the distinction of the expected greater predisposition to forming hexagonal order at the local scale.

## Discussion

The close relationship between (effective) hyperuniformity and the Quantizer problem and the dependence on dimension should be seen in the broader context of related problems. The Kelvin problem^27,46^ of the least-area partition of space into cells of equal volume shows a similar behaviour for sheared monodisperse soap froths: 2D foam is far more prone to forming hexagonal optimal structures; in 3D, no ordering trend is observed, neither in coarsening^47^ nor in sheared foams^48^. Of a similar nature is the packing of spheres. While the 3D system shows Bernal’s celebrated random close packing limit^49^, 2D monodisperse systems do not exhibit a maximal random packing fraction.

We have described how, surprisingly, the analogous concepts for the Quantizer problem lead to the emergence of disordered effectively hyperuniform and universal states. Do different minimisation techniques converge to the same local minima? Are these universal effectively hyperuniform amorphous states unique to the Quantizer problem and its immediate relatives or do they appear in more general repulsive many-particle systems?

Further extensive studies of the corresponding energy landscapes and structural characteristics are necessary to identify the essential properties of the energy landscape that cause this universal phenomenon. Besides properties of the particle interaction, a crucial requirement for the universal behaviour is the complete amorphousness of the system. Local crystallites lead to strong energy barriers, causing distinctive structural differences in the final configurations.

Under which conditions can a successive purely local optimisation converge to amorphous inherent structures that are hyperuniform? Are such configurations in general only effectively hyperuniform? In the broader concept of materials science, our work on the Quantizer energy warrants the search for disordered (effectively) hyperuniform phases in all systems where BCC symmetry phases occur, from amphiphilic micellar phases in copolymers^50^ and lipids^51^ and self-assembling diblock-copolymers^52,53^ to arthropod nanostructures^54^, or in systems that otherwise prefer similarly-sized ‘sphere-like’ cells. Furthermore, while defined through structural organisation at large length scales, hyperuniformity is frequently observed in systems with short-range order, as noted in the context of photonic materials^14–16^. Our work points to the possibility that centroidal Voronoi diagrams, which tend to homogenise local point-point distances, may be a link between short- and long-wavelength properties of these remarkable phases of matter. It raises the question whether there is an intrinsic connection between the Quantizer problem and hyperuniformity. The existence of this link could be further substantiated by exploring the similarities between the local dynamics of Lloyd iterations and the dynamics in other systems represented by Voronoi tessellations. Natural candidates for this behaviour are solidified cellular structures in arthropods^55^ and epithelia^56^, growing and dividing cell arrays^57^, soft colloidal particles under compression^58^, or related glass-forming systems^59,60^. In particular, it would be interesting to explore in future work whether there is a link between CVTs and vertex models for biological tissues^61–63^, even though they involve different energy functionals that minimise fluctuations in the volumes and isoperimetric ratios of the cells and that generally have different ground states. Near hyperuniformity has recently been reported for vertex models^63,64^. While these models differ distinctly from CVTs for small values of the isoperimetric ratio, it might be more promising to compare them in the limit of large isoperimetric ratios, in particular to investigate the universality of a conjectured minimal surface area structure for cellular structures controlling a rigidity transition^62^. It is hence an exciting possibility that the universal effectively hyperuniform amorphous states, as identified in this work, may be found in the thermodynamic phase behaviour across a broad spectrum of systems, including soft materials, active matter and biological tissues.

## Methods

### Lloyd iterations and initial configurations

Lloyd’s procedure is an iterative algorithm for the evolution of sets of points. In each iteration, the Voronoi cells for the point set are constructed, and each generating point is replaced by the centre of mass **c**_*i*_ of its Voronoi cell, which is defined as ${\mathbf{c}}_i: = \frac{1}{{V(C_i)}}{\int}_{C_i} {\mathbf{x}}{\kern 1pt} d{\mathbf{x}}$; *V*(*C*_*i*_) is the volume of the Voronoi cell *C*_*i*_. In 3D the Voronoi cells were constructed by voro++^65^; for the details of the simulation procedure, see Supplementary Note 7.

The data in Figs. 3 and 4, as well as in Supplementary Figs. 2–9 and the Supplementary Table 2 is obtained by at least 10^4^ iterations of Lloyd’s algorithm in 3D and 6.5 × 10^4^ iterations in 2D (the replacement of all generating points by their corresponding Voronoi centres of mass) applied to point sets in simulation boxes with periodic boundary conditions. The number *n* of points per sample varies by almost three orders of magnitude, each sample contains between 5 × 10^2^ and 1.28 × 10^5^ points in 3D and between 5 × 10^2^ and 1 × 10^4^ points in 2D. The process appears to be remarkably independent of the system size. For details on the random number generator see Supplementary Note 1. The initial configurations were generated by the following different stochastic processes (see Supplementary Notes 2 and 3 for further details, parameter values, and references).

Stealthy hyperuniform point process (Stealthy PP): a disordered, statistically isotropic, hyperuniform point configuration, for which the structure factor vanishes not only at infinite wavelength (that is, at *k* = 0) but is zero for all wavelengths above a finite threshold. It is an amorphous state with strong long-range correlations. These point processes can be seen as highly degenerate ground states of special many-particle systems^6^.

Lubachevsky-Stillinger algorithm for jammed static sphere packings (Lubachevsky-Stillinger SphP): Centres of spheres of a static, jammed (mechanically stable) sphere packing at a packing fraction *ϕ* = 0.641 of spheres, below Bernal’s Random Closed Packing limit, obtained by the Lubachevsky-Stillinger algorithm^66^. The hard-core property imposes strong constraints on the sphere configuration. For *ϕ* < 0.648 it is amorphous without any crystallites^44^.

Maximally random jammed state (MRJ SphP): the most disordered among the set of all isotropic, statistically homogeneous, and jammed (mechanically stable) monodisperse hard-sphere packings^42^. It has been previously identified as a prototypical example of disordered hyperuniformity.

Determinantal point process (Determinantal PP), a model for fermions in quantum mechanics and transmitters in wireless networks: All *n*-point correlation functions of the point process are determinants (based on a suitable kernel). It is a repulsive process. Here we have chosen a non-hyperuniform Determinantal PP model, a so-called power exponential spectral model^67^.

Binomial point process (Binomial PP), the ideal gas in the canonical ensemble: A fixed number of points are uniformly and randomly distributed without any correlations between points^41^.

Permanental point process (Permanental PP), the clustering ‘bosonic’ counterpart of the repulsive Determinantal PP: Permutations of a kernel matrix determine the correlation functions. Here, we simulate a strongly anisotropic process with ‘layers’ of clustering points. The local appearance is similar to a Binomial PP.

Hyperplane Intersection Process (Hyperfluctuating PP): the set of all intersection points (of three hyperplanes) of a set of randomly and independently placed and oriented (infinite) hyperplanes, see reference^41^ (p. 313) and references therein. The hyperplanes form a so-called Poisson hyperplane tessellation. The number of planes intersecting the unit ball follows a Poisson distribution. The orientation distribution of the hyperplanes is isotropic. Their distance to the origin forms a 1D Poisson point process on the positive line. The point process formed by the intersections of the random hyperplanes is strongly clustering. The pair correlation function diverges for *r* → 0, see reference^43^. Moreover, the point process is the opposite of hyperuniform. Its ‘hyperfluctuating’ or ‘anti-hyperuniform’ point pattern exhibits structural features on all length scales^42^. The density fluctuations of the intersections quickly grow for large scales; the number variance grows faster than the volume of the observation window^43^; the structure factor diverges for *k* → 0.

Random sequential addition (RSA SphP), a random packing of hard spheres: spheres (or disks in 2D) arrive subsequently, independently and at completely random coordinates^41^. If such a sphere intersects a sphere that was inserted earlier, the new sphere is rejected. Thus, impenetrable spheres (or disks) are subsequently and randomly placed into the system until no sphere can be inserted any more (saturation limit).

Thomas point process (Thomas PP), a clustering of offsprings: Starting with points (sometimes called ‘parent points’) that are generated by a Poisson point process (that is, a completely random snapshot of an ideal gas in the grand-canonical ensemble), each parent point is replaced by a cluster of ‘offsprings’, whose number follows a Poisson distribution and whose coordinates are independent of each other and follow a normal distribution.

Within the first hundred iterations, Lloyd iterations converge quickly for all considered initial configurations. In particular, we find the convergence rate of the energy, measured in the number of Lloyd iterations, to be approximately independent of the number of particles in the system. It is particularly noteworthy that at the beginning Lloyd iterations converge fast even for the hyperfluctuating process. This emphasises its ability to level hierarchical structures, which appear across length-scales. All error bars in this work represent the standard error of the mean. The latter is estimated by the corrected sample standard deviation (using Bessel’s correction).

### Energy density and rescaled dimensionless Quantizer energy

The ‘absolute single cell energy’ *E*(*C*_*i*_, **z**_*i*_) of cell *C*_*i*_ according to Eq. (1) can be expressed as the trace of a so-called second-rank Minkowski tensor^44,68^ from integral geometry. The second-rank Minkowski tensor $W_0^{2,0}$ of a body *K* describes the second moment of the mass distribution in a homogeneous body, $W_0^{2,0}(K) = {\int}_K {\mathbf{x}} \otimes {\mathbf{x}}{\kern 1pt} d{\mathbf{x}}$, where **x** is a position vector with respect to the origin and **x** ⊗ **x** is a second rank tensor with components (**x** ⊗ **x**)_*ij*_ = *x*_*i*_*x*_*j*_. If the generating point of the cell is considered as the origin, the energy is the trace of the Minkowski tensor: $E(C_i,\mathbf{0}) = {\it tr}\left[ {{\it W}_0^{2,0}({\it C}_{\it i})} \right]$ (‘moment of inertia’).

The ‘dimensionless (rescaled) total energy’ of a system with *n* particles in a simulation box Ω ⊂ **R**^*d*^, with *d* = 2 for the planar and *d* = 3 for the spatial system, is given by

2 $$e_t: = \frac{{n^{2/d}}}{{d|{\mathrm{\Omega }}|^{1 + 2/d}}}\mathop {\sum}\limits_{i = 1}^n E(C_i,{\mathbf{z}}_i).$$

It agrees with the definition of the ‘scaled dimensionless Quantizer energy (or error)’^30^, as well as with the mean value of the dimensionless energy of the single cells, see Supplementary Fig. 5:

3 $$\tilde E: = \frac{{\rho ^{1 + 2/d}}}{d}\langle E(C_i,{\mathbf{z}}_i)\rangle ,$$

where *ρ* is the intensity, and 〈…〉 denotes the ensemble average. Note that the dimensionless energy of a single cell depends on the size of the cell.

To compare the shape of the cells irrespective of their volumes, we define the energy density *e* of a single cell as the absolute energy *E* of the cell rescaled by the volume *V* of the cell:

4 $$e: = \frac{1}{{d{\kern 1pt} V^{1 + 2/d}}}E.$$

In general, the average of *e* differs from the global scaled dimensionless Quantizer energy (except for monodisperse tessellations).

The ground state (i.e., minimal energy state) of the ‘Quantizer energy’ in 3D is believed to be the crystalline body-centred cubic (BCC) lattice and in 2D it is the hexagonal lattice^30,33^. Interestingly, the currently known most hyperuniform configurations are the BCC and triangular lattice in 3D and 2D^1^.

The dimensionless total energy in 2D is for the hexagonal lattice *e*_*t*_ ≈ 0.080187 and for the square lattice *e*_*t*_ = 1/12. In 3D, the energy of the BCC lattice is *e*_*t*_ ≈ 0.078543, for the face-centred cubic (FCC) lattice *e*_*t*_ ≈ 0.078745, for the simple cubic (SC) lattice *e*_*t*_ = 1/12, and for the hexagonal Bravais lattice it is *e*_*t*_ ≈ 0.081236.

Starting from the disordered initial conditions such as the Binomial or Hyperfluctuating PP with energies ≈ 0.1159 and ≈ 0.3523, Lloyd’s algorithm converged in 3D to a local minimum^40,69^ with total energies ≈ 0.07922 that vary from 0.07920 to 0.07922 (that is by about 0.01%) between samples and different initial conditions. In 2D, the average final energy of the converged configurations is ≈0.08080(6). The relative decrease of the energies for new iterations was less than 10^−8^ for the final configurations in 3D and less than 10^−14^ in 2D.

The essence of the cell energy analysis in the main text and in Figs. 3c,d and 4a,b is reflected in the energy density *e* from Eq. (4). The inset of Fig. 3d shows that a significant fraction of cells (3%) has lower energy densities than the globally optimal BCC structure–despite comparable volume, as shown in Supplementary Fig. 5, which discriminates between cell energies and cell volume.

In 2D, the fraction (0.2%) of cells with energy densities lower than the hexagonal optimal cell is an order of magnitude smaller than in 3D. Moreover, these cells are exclusively larger than the average cell, in contrast to 3D, see Supplementary Fig. 5. Note that Fig. 3d also illustrates the near-degeneracy of several crystalline configurations, with BCC, HCP, FCC all very close to one another relative to the width of the distribution.

### Stable crystallites

It is has been rigorously proven that at a constant number of points and simulation box size (i.e., constant average Voronoi cell volume), the configuration where the points form a hexagonal lattice in 2D or a BCC lattice in 3D has the lowest overall energy value among all the lattices in 2D or 3D, respectively^30,33^. It is even proven that the hexagonal lattice is the ground state with respect to all possible disordered or quasicrystalline states. The same is conjectured for the BCC lattice among all ordered and disordered spatial systems.

There has been substantial work addressing the sensitivity of Lloyd’s algorithm to structural noise in the vicinity of the locally-optimal crystalline configurations, for more details see Supplementary Note 4, where we have also applied Lloyd iterations to slightly perturbed lattices in 2D and 3D.

If local crystallites of the stable lattices, like FCC or BCC crystals are inserted into an amorphous system, these Voronoi cells do not remain unchanged due to the random environment, but their deformation costs energy in the system. Therefore, local crystallites set up energy barriers that bar the way to the “universal” minimum. However, they do not cause a global crystallisation of the system. An amorphous system with isolated crystallites (e.g., the Lubachevsky-Stillinger system at a packing fraction *ϕ* = 0.659581) still converges to an effectively hyperuniform, disordered CVT with similar but slightly more pronounced features, compared to the universal CVT that is obtained for purely stochastic initial conditions. The strong energy barriers indicate the thermodynamic stability of these states. The BCC and FCC lattices correspond to deep minima in the energy landscape, but they have only a very limited range of attraction in the energy landscape of Voronoi tessellations.

Within the variety of initial configurations that we have analysed, we found statistically significant deviations from the universal effectively hyperuniform and amorphous states only if the initial conditions were partially crystalline. A formalisation of the finding of “universality” for amorphous initial conditions requires a rigorous quantification of the “sufficient degree of randomness”, for which the system converges to the universal effectively hyperuniform and amorphous states. It should exclude partially crystalline hard-sphere packings, but include stealthy hyperuniform systems (perhaps even perturbed lattices if the perturbations are strong enough).

### Minkowski structure metrics

The Minkowski structure metrics^68^ can sensitively characterise the shape of any convex body; for more details, see Supplementary Note 5. Because a Voronoi cell *C* is convex, it is uniquely identified by the so-called surface Minkowski tensors $W_1^{0,s}$, which describe the ‘density function’ $\rho _s^m(C)$ of the outer normal vectors of *C*. The latter can be decomposed into spherical harmonics $Y_s^m$:

5 $$\rho _s^m(C) = \sqrt {\frac{{4\pi }}{{2s + 1}}} \frac{{\mathop {\sum}\limits_k A_kY_s^m({\mathbf{n}}_k)}}{{\mathop {\sum}\limits_k A_k}},$$

where *A*_*k*_ are the surface areas of the cell faces *k* = 1,2,… and **n**_*k*_ the outer normal vectors. This irreducible representation of the Minkowski tensors $W_1^{0,s}$ provides rotational invariants, the ‘Minkowski structure metrics’:

6 $$q_s(C): = \mathop {\sum}\limits_{m = - s}^s |\rho _s^m(C)|^2,$$

7 $$w_s(C): = \mathop {\sum}\limits_{m_1 = - s}^s \mathop {\sum}\limits_{m_2 = - s}^s \mathop {\sum}\limits_{m_3 = - s}^s \left( {\begin{array}{*{20}{c}} s & s & s \\ {m_1} & {m_2} & {m_3} \end{array}} \right)\rho _s^{m_1}(C)\rho _s^{m_2}(C)\rho _s^{m_3}(C),$$

where the parenthesised array is Wigner’s 3*j* symbol.

The Minkowski structure metrics *q*_*s*_ and *w*_*s*_ characterise a convex polyhedron ignoring its scale, position and orientation. Because they are continuous functionals of the convex cell, they robustly identify local crystalline motifs, e.g., small clusters of BCC or FCC Voronoi cells^44^. If there were even small clusters of BCC or FCC Voronoi cells in the 3D CVTs, they would be sensitively detected by peaks in the probability density distributions of *q*_*s*_ and *w*_*s*_, see Supplementary Fig. 3. For software of Minkowski tensors in 2D and 3D and an interactive online tool for ‘Irreducible Minkowski Tensors’ in 2D, see the website https://www.morphometry.org.

### Effective universality

In 3D, the structure factor of the final configurations collapsed for all fully stochastic initial conditions that we studied to the same curves within statistical error bars. The same applies to the energy (density) distributions (see Fig. 3 and Supplementary Fig. 6) and the Minkowski structure metrics (see Supplementary Fig. 3).

In 2D, the final configurations are also essentially independent of the initial conditions. However, subtle changes in the sizes of local crystallites and borders between them can appear for extreme initial conditions, like ones with strong-clustering behaviours. For example, the distribution of the energy density is a compound of a “peak” corresponding to the slightly distorted regular hexagons and a “shoulder” determined by the cells in borders between local crystallites; see Fig. 4 and Supplementary Fig. 6. We expect that the latter can slightly vary, e.g., for initial conditions with strong-clustering behaviours. Thus the domain size statistics can, to some degree, be tuned by the initial process, and slight large-scale density fluctuations could remain in the system or only vanish very slowly using Lloyd iterations.

### Structure factor and *τ* order metric

The structure factor *S*(**k**) is defined via the Fourier transformation ${\cal F}$ of the pair-correlation function *g*_2_, $S({\mathbf{k}}): = 1 + \rho {\cal F}[g_2 - 1]({\mathbf{k}})$, where *ρ* is the intensity of the point process and *g*_2_(**r**) is the ratio of the probability density to find a particle at position **r** (given another point at the origin) and the intensity *ρ*; see Supplementary Note 1. The absolute value of the wave vector **k** is given by *k* = 2*π*/*λ*, where *λ* is the wavelength of the plane wave. Here, the structure factor is estimated from a sample of *N* points at positions **x**_*j*_ by the ‘scattering intensity’ ${\cal S}(k): = \frac{1}{N}\left| {\mathop {\sum}\nolimits_{j = 1}^N e^{ - i{\mathbf{k}} \cdot {\mathbf{x}}_j}} \right|^2$, which converges for *k* > 0 in the thermodynamic limit to the structure factor^42^.

If particles are arranged on a lattice, *S*(*k*) exhibits sharp peaks, called Bragg peaks. Figure 3b shows that *S*(*k*) of the CVTs found in this study cannot be associated with a crystal with periodic order, for neither the initial nor the final point configurations. In particular, the configurations are clearly distinct to the body-centred cubic (BCC) point pattern. So *S*(*k*) also cannot be interpreted as a result of peak-broadening due to fluctuations of the point positions around the vertices of a BCC lattice.

The order metric^6^ *τ* is defined for an isotropic system as:

8 $$\tau : = \frac{{\omega _d}}{{(2\pi )^d}}{\int}_0^\infty k^{d - 1}\left[ {S(k) - 1} \right]^2{\kern 1pt} dk,$$

where the unit of length is chosen such that the intensity is unity, *d* is the dimension, and *ω*_*d*_ is the surface area of the *d*-dimensional unit ball (with *ω*_2_ = 2*π* and *ω*_3_ = 4*π*). This scalar quantity measures the ‘degree of short- and long-range correlations’ in a system^6^. It vanishes for the uncorrelated Poisson or Binomial point process. It diverges for a crystal or the large-scale clustering produced by the intersections of random hyperplanes (Hyperfluctuating PP), as well as for point processes with deterministic (that is, fixed) nearest-neighbour distances, like strictly jammed hard-sphere packings.

For finite simulation boxes, the order metric can be studied as a function of the upper limit of integration:

9 $$\tau (K): = \frac{{\omega _d}}{{(2\pi )^d}}{\int}_0^K k^{d - 1}\left[ {S(k) - 1} \right]^2{\kern 1pt} dk.$$

The Supplementary Table 2 lists the values of the *τ* order metric for the initial and final configurations in both 3D and 2D. While the initial values vary from zero to infinity, the structure factor of the final configurations agrees as a function of the wavenumber. Therefore, also the values of the order metric $\tau$=31(1) in 3D and $\tau$=40(1) in 2D agree within statistical errors, which further confirms and quantifies the universality of the structure factor.

### Hyperuniformity analysis of finite samples

Within a few hundred iterations, even a hyperfluctuating system quickly converges under Lloyd iterations to an effectively hyperuniform system, for all system sizes studied here. The final configurations are almost stealthy hyperuniform with *S*(*k*) < 3 × 10^−3^ for *k*<3 at unit number density, see Fig. 3. Here we provide a short overview of the hyperuniformity analysis of our finite samples, for more details, see Supplementary Note 8.

Recall that for all practical intents these final configurations essentially appear to be hyperuniform, but are clearly not strictly hyperuniform. The question, whether ideal amorphous Voronoi tessellations where the centroids coincide exactly with the Voronoi centre might be strictly hyperuniform, cannot be rigorously answered by our numerical study.

There are four main limitations: (1) a finite number of iterations of Lloyd’s algorithm, which cannot alter the asymptotic behaviour of the number variance; it is worsened by the observation that local configurations can seem to ‘freeze’; (2) finite system sizes effects of Lloyd iterations via the boundary conditions^70^, which cannot be avoided because Lloyd iterations propagate through the entire system, (3) finite system size effects of the structure factor analysis (hyperuniformity is defined in an infinite system), and (4) strong, non-Gaussian statistical fluctuations of the structure factor at small wavenumbers.

Because of the third limitation, we only consider wavenumbers larger than 4.5 × *k*_*min*_, where *k*_*min*_ is for each simulation box the minimal value of the wavenumber. The only exceptions are the samples of the Stealthy and Determinantal PP, where we have to consider wavenumbers *k* > 1.5*×k*_*min*_ because of the small system sizes.

How close an experimental or simulated sample is to perfect hyperuniformity, can be quantified by the hyperuniformity index *H*, which is defined as the ratio of $\hat S(0)$, a linear extrapolation of the structure factor to *k* = 0 using a least-square fit, and *S*(*k*_*peak*_), the largest peak of the structure factor^42^:

10 $$H: = \frac{{\hat S(0)}}{{S(k_{peak})}}.$$

A finite sample is typically ‘deemed to be nearly hyperuniform’ if *H* ≤ 10^−3^, see reference^42^ and Supplementary Table 1. Note that for the reasons explained above and in Supplementary Note 8, the linear extrapolation is chosen for simplicity and not based on a model selection, and we use only wavenumbers larger than 4.5 × *k*_*min*_ (or *k* > 1.5×*k*_*min*_ for the samples that originated from the Stealthy or Determinantal PP).

For the 3D CVTs, our estimates $\hat H$ are within statistical errors consistent with *H* = 0, that is, with perfect hyperuniformity, with mean values of $\hat H$ smaller than 10^−4^. For the 2D CVTs, there is a slight (but statistically significant) non-monotonic behaviour of the structure factor for small wavenumbers. Whether freezing effects of the almost hexagonal cells prevent the system from becoming hyperuniform remains an open question. If wavenumbers *k* < 1.5, which are strongly influenced by this effect, are omitted, and the structure factor is extrapolated by a least-square fit of a line to the approximately linear regime *k* ∈ [1.50, 4.50], then the estimates of *H* are smaller than 10^−4^ at unit density.

In mathematics, there is recent interest in ‘rigidity phenomena’, where the number of points within a finite observation window or even their positions are determined by the points outside of the observation window^5^. The strong constraint of a CVT, where each Voronoi centre coincides exactly with the centre of mass, motivates the question whether CVTs are in the mathematical sense strongly rigid.

### Code availability

All code used during the current study are available from the corresponding authors on reasonable request.

## Supplementary information

Source Data

Supplementary Information

Description of Additional Supplementary Files

Supplementary Movie 1

## Data Availability

All data generated or analysed during this study and the source data underlying Figs. 1–4 are included in this published article (and its supplementary information files), see Supplementary Data 1.
