# Supplementary material for: Universal hidden order in amorphous cellular geometries
Source: Nat Commun. 2019 Feb 18;10:811. doi: 10.1038/s41467-019-08360-5 (PMC6379405; doi:10.1038/s41467-019-08360-5)
Supplement: Supplementary file 2 — Supplementary Information [file 41467_2019_8360_MOESM2_ESM.pdf]

Supplementary Information:
Universal hidden order in amorphous cellular geometries

Michael A. Klatt[1,2,*,†], Jakov Lovrić[3,4,5,†], Duyu Chen[6], Sebastian C. Kapfer[7,*], Fabian M. Schaller[7,1], Philipp W.A. Schönhöfer[4,7], Bruce S. Gardiner[4,8], Ana-Sunčana Smith[3,5,*], Gerd E. Schröder-Turk[4,7,9,*,‡] & Salvatore Torquato[10,*,‡]

[1] *Karlsruhe Institute of Technology (KIT), Institute of Stochastics, Englerstr. 2, 76131 Karlsruhe, Germany*
[2] *Department of Physics, Princeton University, Princeton, New Jersey 08544, USA*
[3] *Division of Physical Chemistry, Ruđer Bošković Institute, Bijenička 54, 10 000 Zagreb, Croatia*
[4] *Murdoch University, School of Engineering and Information Technology, 90 South St, Murdoch WA 6150, Australia*
[5] *PULS Group, Department of Physics and Interdisciplinary Center for Nanostructured Films, Friedrich-Alexander-Universität Erlangen-Nürnberg, Cauerstraße 3, 91058 Erlangen, Germany*
[6] *Department of Chemistry, Princeton University, Princeton, New Jersey 08544, USA*
[7] *Institut für Theoretische Physik, Friedrich-Alexander-Universität Erlangen-Nürnberg, Staudtstr. 7, 91058 Erlangen, Germany*
[8] *The University of Western Australia, School of Computer Science and Software Engineering, 35 Stirling Highway, Crawley WA 6009, Australia*
[9] *Department of Applied Mathematics, Research School of Physical Sciences and Engineering, The Australian National University, Canberra 0200 ACT, Australia*
[10] *Department of Chemistry, Department of Physics, Princeton Institute for the Science and Technology of Materials, and Program in Applied and Computational Mathematics, Princeton University, Princeton, New Jersey 08544, USA*
[†,‡] *These authors contributed equally to this work.*

Contents

1 Supplementary Note: Point processes **3**

2 Supplementary Note: Initial configurations in 3D **4**

3 Supplementary Note: Initial configurations in 2D **8**

4 Supplementary Note: Perturbed lattices and stable crystallites **10**

5 Supplementary Note: Minkowski structure metrics **11**

6 Supplementary Note: Quantizer problem as a classical system of interacting particles **12**

7 Supplementary Note: Centroidal Voronoi Tessellations **13**

8 Supplementary Note: Hyperuniformity analysis for finite samples **14**

Supplementary Figures **18**

Supplementary Tables **27**

Supplementary References **28**

1 Supplementary Note: Point processes

Mathematically speaking, the initial configurations analysed in the main text are realisations of so-called point processes.

Point processes model particles like fermions, bosons, or atoms in liquids or granular matter, but also nodes in telecommunication networks, trees in forests or galaxies, to name just a few applications; for example, see references 1–3 and references therein. Probability theory and stochastic geometry offer a rigorous definition and analysis of these point processes[4,5] and allows for a statistical inference for point patterns[6–8].

A point process is a random collection of (at most countably many) points[4]. More precisely, it is a mapping from the element of a probability space to a countable subset of the underlying space. Here, we only consider point processes in the d-dimensional Euclidean space $\mathbf{R}^d$, specifically for $d = 2$ and $d = 3$. Note, however, that the mathematical definition of a point processes is applicable to much more general spaces, e.g., to the space of convex bodies.

Alternatively, a point process can be defined as an integer-valued random measure, which is a measure depending on a parameter that belongs to a probability space[5]. The measure can be thought of as a counting measure η that assigns to a Borel set $B \subset \mathbf{R}^d$ the number of points it contains, which is denoted by $\eta(B)$.

Moreover, we only study locally finite point processes: A bounded set can only contain a finite number of points. Our point processes are simple, that is, two points in the process cannot occupy the same site (technically known as multiplicity equal one). Finally, the point processes in this study are diffuse, i.e., for all positions x the probability to find a particle at exactly this position is equal to zero.

Note that although the point processes considered here are stationary (i.e., statistically homogeneous, the distributions do not depend on the choice of the origin), this is not a necessary condition for the convergence of Lloyd iterations to the universal configurations as reported in this study. In fact, the samples of the permanental process can also be thought of as realisations of an inhomogeneous Poisson point process (see below). Moreover, when we construct the Voronoi diagram using periodic boundary conditions for point processes that have been simulated using *minus sampling boundary* conditions (that is, simulating a subset of the infinite model) the tessellations become, strictly-speaking, non-stationary.

For a stationary point process, the intensity ρ (also called number density) is the expected number of points $\mathbf{E}[\eta([0,1]^n)]$ that fall into the unit cube, where the expectation $\mathbf{E}$ corresponds to the ensemble average. To compare different point processes at the same length scale, we choose for each sample the unit of length such that the intensity is equal to unity. More precisely, we choose it

such that the estimated intensity, given by the ratio of the total number of points and the size of the observation window, is equal to one. The length scale is adjusted for each sample separately so that the final configurations are compared at the same number density. Otherwise strong fluctuations, e.g., in the hyperfluctuating system, would not be compensated in our finite samples due to the periodic boundary conditions.

The n-point correlation functions g_n of a point process satisfy

$$\mathbf{E}[\eta(\mathbf{B_1})\cdots\eta(\mathbf{B_n})] = \int_{B_1\times\ldots\times B_n} \rho^n g_n(x_1,\ldots x_n) d(x_1,\ldots x_n), \tag{1}$$

for all pairwise disjoint Borel sets $B_1, \ldots B_n \subset \mathbf{R}^n$. For a finite point pattern, an intuitive interpretation of $\rho^n\, g_n(x_1,\ldots x_n)d(x_1,\ldots x_n)$ is the probability to find n points in the infinitesimal neighbourhoods of $x_1,\ldots x_n$.

In our simulations of random point patterns, we use the MT19937 generator[9] (known as "Mersenne Twister") to generate random numbers, as implemented in the SciPy library[10] the statistics software R[11], the GNU Scientific Library[12], and the Boost Libraries[13].

2 Supplementary Note: Initial configurations in 3D

In 3D, we use the following point processes as initial conditions for Lloyd iterations. Their realisations, that is, the actual point patterns, range from rather structured to highly irregular point patterns, see Supplementary Fig. 1.

A Stealthy Hyperuniform Point Process (Stealthy PP) is a point process designed to produce hyperuniform configurations, for which the structure factor vanishes not only at an infinite wavelength (that is, at $k = 0$) but at all wavelengths above a finite threshold (that is, for a finite range of wavenumbers $k < k_0$ where $k_0 > 0$). This is trivially fulfilled for a crystal with only Bragg peaks in the structure factor. However, there are also disordered, statistically isotropic stealthy hyperuniform point processes. Their point patterns are examples of highly degenerate ground states of many-particle systems with special pair-potentials[14].

To generate disordered "stealthy" point configurations, we employ the following stealthy interaction potential[15,16]:

$$\Phi(\mathbf{r}^N) = \frac{N}{2v_F}\sum_{0<\mathbf{k}<K} S(\mathbf{k}) + \Phi_0 = \frac{1}{v_F}\sum_{i<j}\sum_{0<\mathbf{k}<K} \exp(i\,\mathbf{k}\cdot\mathbf{r}_{ij}),$$

where N is the number of points, v_F is the volume of the simulation box, $\mathbf{k}$ is the wave vector, the constant K specifies the radius of the "exclusion" sphere around the origin within which the

structure factor $S(\mathbf{k})$ is constrained to zero in the ground state; $\Phi_0 = [N(N-1) - \sum_{0<\mathbf{k}<K} N]/(2v_F)$ is a constant independent of the particle positions $\mathbf{r}^N$.

Let M be the number of independent $\mathbf{k}$ points within the exclusion sphere; then the parameter $\chi = \frac{M}{d(N-1)}$ determines the degree to which the ground states are constrained and therefore the degeneracy and disorder of the ground states[15,16]. For $\chi < 0.5$, the ground states are typically disordered and uncountably infinitely degenerate. As χ increases, the short-range order of the system increases. At $\chi = 0.5$, the system transitions into crystalline states. For $\chi < 0.5$, as the grounds are degenerate, there are multiple ways to assign different weights (i.e., probabilities) to different sets of ground states. We are in particular interested in the "entropically favoured ground states"[15,16], which are drawn from the canonical ensemble probability distribution function defined by $P(\mathbf{r}^N) = \exp[-\beta\Phi(\mathbf{r}^N)]/Z$, where β is the inverse temperature, and $Z = \int \exp[-\beta\Phi(\mathbf{r}^N)]d\mathbf{r}^N$ is the partition function.

We generate entropically favoured ground states of stealthy potentials using the same protocol as that in reference 16. This protocol involves performing molecular dynamics (MD) simulations at a very low temperature ($\beta = 1 \times 10^6$ in dimensionless units), taking snapshots periodically, and performing a local energy minimisation starting from each snapshot. Because the MD temperature is sufficiently low, the snapshots before energy minimisation are already very close to ground states. Therefore, the ground states produced by the subsequent energy minimisation closely follow the canonical distribution in the zero-temperature limit. Specifically, we generate–in non-orthotropic observation windows with periodic boundary conditions–twelve configurations at $\chi = 0.494$ with $N = 500$ points.

Lubachevsky-Stillinger Sphere Packings (Lubachevsky-Stillinger SphP) are jammed, non-equilibrium packings of monodisperse, frictionless, hard spheres, that is, balls that are impenetrable but do not interact otherwise[17]. Here, we study the point process formed by the centres of these spheres. The static sphere packings were obtained by the Lubachevsky-Stillinger algorithm with data set as in reference 18. We simulated a sample with 40000 spheres at a global packing fraction $\phi = 0.640639$, which is the volume fraction occupied by the non-overlapping spheres. The Lubachevsky-Stillinger algorithm was also used to generate amorphous configurations with isolated crystalline domains ("crystallites") at $\phi = 0.659581$, see Section 4. Our simulation parameters follow reference 18 using the Lubachevsky-Stillinger implementation from reference 19.

Maximally Random Jammed Sphere Packings (MRJ SphP) are the most disordered among the set of all isotropic, statistically homogeneous, and jammed (mechanically stable) monodisperse hard-sphere packings[20,21]. More precisely, the MRJ state minimises some order metric among all strictly jammed packings, that is, no collection of particles can be moved without deforming the system boundary. The MRJ state is hyperuniform[22]; for an overview of geometric properties of the

MRJ sphere packings and their Voronoi cells, see references 23, 24.

The packings are generated in a deformable periodic cell by the Torquato-Jiao (TJ) sphere packing algorithm[25], which solves a sequence of linear programs iteratively and thereby densifies the system subject to locally linearized nonoverlap constraints. The data sets are generated in the same manner as those in references 25, 26 and consist of 1015 packings each containing 2000 spheres.

The Determinantal Point Process (Determinantal PP) models fermions in thermal equilibrium (distributed in the Euclidean space)[27]. It is a strongly repulsive process at short distances, which leads to a more regular point pattern than that of a completely uncorrelated Binomial point process. The positions of the points can be interpreted as an ideal (error-free) detection of the fermions at a given time[27]. At zero temperature the determinantal point process provides the exact distributions even for a finite number of particles[28]. The determinantal point process is also used to model transmitters in wireless networks[29].

The mathematical definition is based on a suitable kernel $K : \mathbf{R}^d \times \mathbf{R}^d \to \mathbf{R}$, such that all n-point correlation functions of the point process are determinants of this kernel[30–32]:

$$g_n(x_1, \ldots, x_n) = \rho^{-n} \det \Big(K(x_i, x_j) \Big)_{1 \leq i,j \leq n} . \tag{2}$$

For a survey of its probabilistic properties, see reference 31.

There are hyperuniform Determinantal PP models, like the Ginibre ensemble or Fermi-sphere models[33]. Here we have chosen a non-hyperuniform Determinantal PP model, the so-called power exponential spectral model[32], as an initial condition of Lloyd iterations. The parameters of the model, using the notation in reference 32, are chosen as follows: $\nu = 10$ and $\alpha = (1 - 10^{-4})\alpha_{\max}$, where $\alpha_{\max}$ is the maximal possible value of α (which depends on ν). For these parameters, the structure factor $S(k)$ converges for $k \to 0$ to $S(0) \approx 0.19$. It is clearly non-hyperuniform. The model would only become hyperuniform for $\alpha = \alpha_{\max}$ and $\nu \to \infty$.

We have simulated 20 samples with a software package provided by Ege Rubak[32] written for SPATSTAT[34]; note that by restricting the kernel to a compact subset of the Euclidean space, the Determinantal PP can be restricted to the simulation box. An average of 1000 points per sample where simulated in the unit square using periodic boundary conditions. Subsequently the length scale was rescaled to obtain a unit number density.

The Binomial Point Process (Binomial PP) describes the completely random configurations of snapshots of the ideal gas in the canonical ensemble. The number of points is fixed, but their

coordinates are independently and uniformly distributed in the simulation box[1]. We simulated three samples, each with 128000 points. Because of the independence of the coordinates of the points, the pair correlation function is constant and for large n approximately equal to unity[2]:

$$g_2(r) \equiv 1 - \frac{1}{n}. \tag{3}$$

For $k > 0$, the structure factor $S(k)$ is constant and equal to unity. In the thermodynamic limit, the volume of the simulation box V_s and the number of points diverge, but the number density $\rho = n/V_s$ remains constant.

The Permanental Point Process (Permanental PP) models bosons and is the clustering counterpart of the repulsive determinantal point process[31]. It can also be defined via its n-point correlation functions based on a kernel $K : \mathbf{R}^d \times \mathbf{R}^d \to \mathbf{R}$, but using permutations instead of determinants[4,35]:

$$g_n(x_1, \ldots, x_n) = \rho^{-n} \mathrm{per}\Big(K(x_i, x_j)\Big)_{1 \leq i,j \leq n} = \rho^{-n} \sum_{\sigma \in \Sigma_n} \prod_{i=1}^{n} K(x_i, x_{\sigma(i)}), \tag{4}$$

where Σ_n denotes the group of permutations σ of $\{1, \ldots, n\}$.

The class to which the permanental process that was simulated for this study belongs has the intuitive interpretation of a doubly stochastic Poisson process. First, a random intensity function is simulated. Then, the final point pattern is given by an inhomogeneous Poisson point process (ideal-gas in the grand canonical ensemble) following this density profile.

Among this still huge class of processes, we have chosen random intensity functions that are given by the sum of the absolute values of two realisations of a Gaussian random waves model. The latter is, in turn, defined by the superposition of plane waves with random phases and orientations of the wave vectors[36]. The details of the model used here and the simulation procedure are explained in reference 36. We use minus sampling boundary conditions (that is, the sample is derived by intersecting the simulation box with the infinite point pattern). Two samples were simulated, each containing approximately 8000 points, using the parameters $L = 25$, $|k_i| = 10/L$, $a_w = 1/2$, and $\omega = 0.1$, as explained in reference 36.

By choosing an anisotropic orientation distribution for the wave vectors, we create a strongly anisotropic point pattern with strong density fluctuations in z-direction. Therefore, the structure factor in this direction can become orders of magnitude larger than that in the perpendicular directions. This is indicated by two different symbols for the initial structure factors in Fig. 3 in the main text. However, under Lloyd iterations, even this strongly anisotropic point process converges to the same universal isotropic state as the other disordered point processes.

The Hyperplane Intersection Process (Hyperfluctuating PP) is a point process that originates from a tessellation that is distinctly different from a Voronoi tessellation (which begins with a point pattern for which the Voronoi cells are constructed). The Hyperplanes Intersection Process begins with a tessellation that is formed by a collection of intersecting independent hyperplanes, see reference 1 (p. 313) and references therein.

The so-called Poisson hyperplane tessellation is constructed by intersecting randomly oriented and uniformly distributed hyperplanes in the Euclidean space[37]. More precisely, the number of hyperplanes intersecting a ball follows a Poisson distribution. The distance of each hyperplane that intersects the ball is uniformly distributed between zero and the radius of the ball. This also defines a simple simulation procedure. The points are then obtained by the intersections of three hyperplanes. Here, we have simulated (using minus sampling boundary conditions) a sample containing more than 10500 points and a sample with more than 99700 points.

It is a strongly clustering point process. The pair correlation function $g_2(r)$ diverges for $r \to 0$; for an analytic expression of $g_2(r)$, see reference 38.

Because a hyperplane spans the whole space, it induces infinitely long ranged correlations, which increases the density fluctuations. The Hyperplane Intersection Process is therefore fluctuating qualitatively stronger than a completely random Poisson point process. In this sense, it is the opposite of a hyperuniform process. It is hyperfluctuating[39], that is, there are arbitrary long-range (∞ in an ∞ system) spatial density fluctuations. The variance of the number of points in a d-dimensional spherical observation window of radius R grows like R^{2d-1}, that is, faster than the volume of the observation window (for $d > 1$)[40]. Therefore, the structure factor $S(k)$ diverges for $k \to 0$.

3 Supplementary Note: Initial configurations in 2D

In 2D, we use the following point processes as initial conditions for Lloyd iterations.

The Stealthy Hyperuniform Point Process (Stealthy PP) in 2D follows the same principle as in 3D, described above. In 2D we choose as an initial temperature parameter $\beta = 5 \times 10^5$ in dimensionless units. We generated ten configurations at $\chi = 0.30$ with $N = 500$ points.

Random Sequential Adsorption (RSA) also known as "random sequential addition," "simple sequential inhibition," or "Poisson disc sampling," is an important model, e.g., for the irreversible adsorption or adhesion of proteins or cells at solid interfaces[41]. A particle diffuses above a surface before it attaches to the surface at a random site. Because it can no longer move, it blocks the

adsorptions of other particles in its neighbourhood. This is modelled by RSA which subsequently places spheres on the surface. Specifically, spheres (in 3D) or discs (in 2D) are randomly and sequentially inserted into the simulation box subject to periodic boundary conditions such that they do not overlap with existing spheres in the box that are inserted previously. Once inserted, the spheres do not change their positions. The process is repeated until there is no available space in the box for a new sphere to insert, i.e., the packing achieves saturation. In the mathematics literature, it is known as the saturation limit of the Matérn III process[1]. In simulations, it has been shown that the packing fraction (the fraction of space covered by the spheres) ϕ is 0.547 and 0.384 at saturation in two and three dimensions[42], respectively. Stoyan and Schlather[43] constructed a statistically homogeneous version of RSA (which is well-defined in the infinite volume limit), see reference 1 (p. 240). Here, we generate (using periodic boundary conditions) in 2D ten such packings with around $N = 10^4$ spheres.

The Binomial Point Process (Binomial PP) in 2D follows the same principle as in 3D, described above. In 2D, we simulated and analysed ten samples each containing 10^4 points.

The Thomas Point Process (Thomas PP) is a typical example of a clustering Neyman-Scott process[1,3]. These Poisson cluster processes are also known as centre-satellite processes. Each point in the realisation of a homogeneous Poisson point process is considered to be a "parent point" for a cluster. The intensity of the parent process is denoted by ρ_p. The clusters are independent and follow the same distribution. They have a random number of "children" with mean value $\bar{c}$; these points in a cluster are again independently and identically distributed. If the probability density of the children is isotropic, then the final point process is isotropic, which includes only the children and not the parents. The latter only serve as the centres of the clusters.

The intensity of the Neyman-Scott process is (because of statistical independence) given by $\rho = \rho_p \bar{c}$. For the (modified) Thomas process, studied here, the number of points in a cluster follows a Poisson distribution, and the displacement of a child from its parent follows the isotropic d-dimensional Gaussian distribution with variance σ^2. The pair-correlation function of this process in d dimensions is given by[1,44]:

$$g_2(r) = 1 + \frac{\bar{c}}{\rho}(4\pi\sigma^2)^{-d/2} \exp\left(-\frac{r^2}{4\sigma^2}\right). \tag{5}$$

The parameters $\bar{c}$ and σ determine the "degree of clustering" in the model. Depending on these parameter values, the resulting point patterns can appear similar to a Poisson point process, or they can exhibit strong clustering. Here, we choose $\bar{c} = 10$ and $\sigma = 1$ giving distinctive clustering. To simulate the Thomas process in a simulation box $[0, L)^2$ with periodic boundary conditions, we first draw the number of parent points from a Poisson distribution with mean value $\lambda_p L^2$. The coordinates of the parent points are uniformly distributed in $[0, L)$. For each parent, we then determine

the number of children, which follow a Poisson distribution with mean value $\bar{c}$. Finally, we simulate the coordinates of the children by sampling from the corresponding Gaussian random variables and insert the children subject to periodic boundary conditions. We simulated ten samples each containing on average 10^4 points.

The Hyperplane Intersection Process (Hyperfluctuating PP) is already described in the previous section. In 2D, we simulated and analysed ten samples, which contain between 7×10^3 and 12×10^3 points.

4 Supplementary Note: Perturbed lattices and stable crystallites

It is has been rigorously proven that at a constant number of points and simulation box size (i.e., constant average Voronoi cell volume), the configuration where the points form a hexagonal lattice in 2D or a BCC lattice in 3D has the lowest overall energy value among all the lattices in 2D[45] or 3D[46,47], respectively. It is even proven that the hexagonal lattice is the ground state with respect to all possible disordered or quasicrystalline states. The same is conjectured for the BCC lattice among all ordered and disordered spatial systems. The dimensionless scaled energy e_t takes values of $e_t \approx 0.07854$ for the BCC lattice, $e_t \approx 0.07874$ for the FCC lattice, $e_t \approx 0.07871$ for the A15 configuration and $e_t \approx 0.0791$ for the simple cubic lattice.

There has been substantial work addressing the sensitivity of Lloyd iterations to structural noise in the vicinity of the locally-optimal crystalline configurations[48]. This was done by perturbing point structures in many different ways for distinct boundary configurations. It was shown that BCC, FCC, Z and A15 are stable crystalline lattices but BCC is the most stable[48].

We have also applied Lloyd iterations to slightly perturbed lattices in 2D and 3D. As the initial perfect Bravais lattices we studied the hexagonal or simple square lattice in 2D and the body-centred cubic (BCC), face-centred cubic (FCC), or simple cubic (SC) lattice in 3D. The unit of length is chosen such that the primitive cell has volume 1, corresponding to the number density equal to unity for the random point patterns. We have added small perturbations (drawn from the uniform distribution on $[-\varepsilon, \varepsilon]^3$, where ε varied from 10^{-10} to 0.5) to the positions of each particle and studied the convergence of Lloyd iterations.

As expected, the hexagonal, BCC, and FCC lattices are stable, in the sense that for small perturbations these systems relax under Lloyd iterations to the original perfect lattice configurations. However, if the FCC lattice is perturbed by random displacements up to more than the nearest-neighbour distance, then Lloyd iterations converge–within numerical precision–to the universal disordered effectively hyperuniform Centroidal Voronoi Tessellations (CVTs). For a simple cubic lattice, even very small deviations ($\varepsilon = 10^{-10}$) suffice so that the perturbed lattices converges to

a disordered effectively hyperuniform CVT. The same applies to the simple square lattice in two dimensions. The simple cubic and simple square lattices are unstable lattices. The final configurations are almost indistinguishable from the universal configurations, to which the disordered initial configurations converge. However, if the initial distortions of the cubic lattice are very small (of the order of 10^{-10}), we could detect small yet statistically significant deviations from the universal distribution of energy densities.

If local crystallites of the stable lattices, like FCC or BCC crystals are inserted into an amorphous system, these Voronoi cells do not remain unchanged due to the random environment, but their deformation costs energy in the system. Therefore, local crystallites set up energy barriers that bar the way to the "universal" minimum. However, they do not cause a global crystallization of the system. An amorphous system with isolated crystallites (e.g., the Lubachevsky-Stillinger system at a packing fraction $\phi = 0.659581$) still converges to an effectively hyperuniform, disordered CVT with similar but slightly more pronounced features, compared to the universal CVT that is obtained for purely stochastic initial conditions. The strong energy barriers indicate the thermodynamic stability of these states. The BCC and FCC lattices correspond to deep minima in the energy landscape, but they have only a very limited range of attraction in the energy landscape of Voronoi tessellations.

Within the variety of initial configurations that we have analysed, we found statistically significant deviations from the universal effectively hyperuniform and amorphous states only if the initial conditions were partially crystalline. A formalization of the finding of "universality" for amorphous initial conditions requires a rigorous quantification of the "sufficient degree of randomness", for which the system converges to the universal effectively hyperuniform and amorphous states. It should exclude partially crystalline hard-sphere packings, but include stealthy hyperuniform systems (perhaps even perturbed lattices if the perturbations are strong enough).

5 Supplementary Note: Minkowski structure metrics

Minkowski tensors are sensitive shape descriptors[49,50] that allow for a comprehensive shape analysis of random tessellations[36]; for Minkowski tensors in integral and stochastic geometry, where they are also known as tensor valuations, see reference 51.

The shape of any Voronoi cell C may be succinctly characterised by its Minkowski tensors[49]. In particular, Minkowski's problem[52] uniquely relates any convex polyhedron C to the set of its area-weighted normals $\{\mathbf{A}_k\}$. Here, $k = 1, 2, \ldots$ labels the neighbours of the cell C, $A_k = |\mathbf{A}_k|$ is the interface area between the cell and its k-th neighbour, and the direction $\mathbf{n}_k = \mathbf{A}_k/|A_k|$ is the outer normal vector of the interface. The normals may be conveniently analysed as a density function on

the unit sphere, which is decomposed into spherical harmonics Y_l^m,

$$\rho_l^m(C) = \sqrt{\frac{4\pi}{2l+1}} \frac{\sum_k A_k Y_l^m(\mathbf{n}_k)}{\sum_k A_k}. \tag{6}$$

In this, the spherical harmonic takes its angular parameters φ, θ from the direction $\mathbf{n}_k$. From this decomposition, rotational invariants may be formed which serve as the fingerprints of a particular geometry:

$$q_l(C) = \sum_{m=-l}^{l} |\rho_l^m(C)|^2, \tag{7}$$

$$w_l(C) = \sum_{m_1,m_2,m_3} \begin{pmatrix} l & l & l \\ m_1 & m_2 & m_3 \end{pmatrix} \rho_l^{m_1}(C)\rho_l^{m_2}(C)\rho_l^{m_3}(C). \tag{8}$$

The sums in the latter extend from $-l$ to l, and the parenthesized array is Wigner's $3j$ symbol. The series of q_l and w_l, are characteristic of a particular convex polyhedral shape, ignoring its scale, position or orientation. They thus can be used to identify particular structural motifs occurring in packings[18,53] or general point patterns.

If there were local crystallites in the CVTs, e.g., small clusters of BCC or FCC Voronoi cells, they would be sensitively detected by peaks in the probability density distributions of the Minkowski structure metrics[18].

6 Supplementary Note: Quantizer problem as a classical system of interacting particles

For a classical many-particle system consisting of N identical particles at positions $\mathbf{r}_1, \mathbf{r}_2, \dots \mathbf{r}_N$ in d-dimensional Euclidean space, the total potential energy $\Phi_N(\mathbf{r}_1, \mathbf{r}_2, \dots, \mathbf{r}_N)$ can be resolved into separate one-body, two-body, and up to N-body contributions:

$$\Phi_N(\mathbf{r}^N) = \underbrace{\sum_{i=1}^{N} u_1(\mathbf{r}_i)}_{\text{externalfield}} + \underbrace{\sum_{i<j}^{N} u_2(\mathbf{r}_i, \mathbf{r}_j)}_{\text{twobodyinteraction}} + \underbrace{\sum_{i<j<k}^{N} u_3(\mathbf{r}_i, \mathbf{r}_j, \mathbf{r}_k) + \cdots + u_N(\mathbf{r}^N)}_{\text{higherordercontributions}}, \tag{9}$$

where u_N represents the intrinsic N-body interaction in excess to the interaction energy for $N-1$ particles. Following reference 54, we express the Quantizer problem as such a classical system of interacting particles.

The Quantizer error can be interpreted as an energy functional that assigns a "distortion" energy to each point pattern in d-dimensional Euclidean space. In the limit of an infinite system size, that is, of infinitely many points, the scaled average squared error $\mathcal{G}$ is defined as

$$\mathcal{G} := \frac{1}{d\langle V(\mathcal{V})\rangle^{1+\frac{2}{d}}} \lim_{N\to\infty} \frac{1}{N} \sum_{i=1}^{N} \int_{\mathcal{V}(\mathbf{r}_i)} |\mathbf{x} - \mathbf{r}_i|^2 d\mathbf{x}, \tag{10}$$

where $\mathcal{V}(\mathbf{r}_i)$ is the Voronoi cell with the centre $\mathbf{r}_i$, and $\langle V(\mathcal{V})\rangle$ is the average Voronoi cell volume:

$$\langle V(\mathcal{V})\rangle := \lim_{N\to\infty} \frac{1}{N} \sum_{i=1}^{N} V(\mathcal{V}(\mathbf{r}_i)), \tag{11}$$

where $V(\mathcal{V}(\mathbf{r}_i))$ is the volume of the cell $\mathcal{V}(\mathbf{r}_i)$.

By definition, the error $\mathcal{G}$ is (up to the prefactor $1/d$) equivalent to the second moment of the void nearest-neighbour distance[54] R, that is, of the distance between a random point in the Euclidean space and its nearest-neighbour among the points $\mathbf{r}_1$, $\mathbf{r}_2$, $\ldots \mathbf{r}_N$ in the point process. The complementary cumulative distribution function of R, that is, the probability that a randomly placed sphere of radius R contains no points of the point process, is called the void exclusion probability $E_V(R)$. The scaled error $\mathcal{G}$ can thus be rewritten as

$$\mathcal{G} = \frac{1}{d}\langle R^2\rangle = \frac{2}{d}\int_0^\infty R E_V(R) dR. \tag{12}$$

Using the exclusion-inclusion principle, $E_V(R)$ can be expressed for a single realisation of N points within a large volume v_F by:

$$E_V(R) = 1 - \rho v_1(R) + \frac{1}{v_F}\sum_{i<j} v_2^{\text{int}}(r_{ij}; R) - \frac{1}{v_F}\sum_{i<j<k} v_3^{\text{int}}(r_{ij}, r_{ik}, r_{jk}; R) - \cdots \tag{13}$$

if boundary effects can be neglected (because N is sufficiently large).

In analogy to equation (9), we can interpret equation (13) as an expansion in terms of n-body contributions. The $(n+1)$st term is a sum over the intrinsic n-body interactions v_n^{int}. The second term in equation (13), which is a sum over one-body contributions, is independent of the point configuration. The first term is a trivial additive constant offset in the energy.

The void exclusion probability of the different initial configurations has a strongly different behaviour for large R. It decays exponentially for the Binomial point process, but it has a compact support for the Stealthy PP or the MRJ SphP, that is, there is an upper bound on the allowed values of R. This corresponds in this picture of a many-particle system to differently long-ranged interactions w.r.t. the Quantizer energy.

7 Supplementary Note: Centroidal Voronoi Tessellations

Quantization is a common problem in signal compression in information theory[55], numerical integration[56], mathematical models in economics[57], cellular biology[58], and for the territorial behaviour of animals[59–61]. The adjustment models of territory by Hasegawa and Tanemura from 1976 and 1980 are using variations of Lloyd's algorithm[62–64]. Lloyd iterations have also been used to

produce optimal photonic crystals[65]. The optimal quantization of Centroidal Voronoi Tessellations (CVT) is important to a variety of seemingly unrelated topics[61,66].

In our study, we have implemented Lloyd iterations and analysed the CVTs using the following software: QHULL[67], VORO++[68,69], and MATLAB[70–72] for constructing the Voronoi tessellations; PAPAYA[73], KARAMBOLA[49,73–75], POMELO[76], and the SCIPY library[10] were used for the geometrical analysis.

To construct the Voronoi diagram of the point pattern inside a simulation box, we apply periodic boundary conditions. While no subset of our point pattern exhibits periodicity, the whole system is studied on the torus, and the CVTs are, strictly-speaking, so-called Periodic Centroidal Voronoi Tessellations (PCVTs). For a mathematical study of PCVTs and a first analysis of the energy landscape for up to 20 particles, see reference 77.

8 Supplementary Note: Hyperuniformity analysis for finite samples

The structure factor converges under Lloyd iterations quickly to an effectively hyperuniform system for all system sizes studied here, see Fig. 3. The algorithm can reduce $S(k)$ by orders of magnitude within a few hundred iterations of a strongly repulsive local optimisation. In our final configurations, the values of $S(k) = \mathcal{O}(10^{-3})$ for small k are similar to the values observed for the Maximally Random Jammed (MRJ) packings deemed hyperuniform[22]. Here we discuss the limitations of our numerical analysis w.r.t. a rigorous confirmation or negation of strict hyperuniformity, and we provide a quantitative analysis confirming the effective hyperuniformity within the accuracy of this numerical study.

For an ergodic system, an ensemble average $\langle . \rangle$ over different realisations with a fixed window is equivalent to a spatial average over the positions of the window for a fixed realisation. The latter allows the definition of hyperuniformity for a realisation (or configuration/sample) rather than for the process that generates it. Strictly speaking, hyperuniformity is a property of the infinite system (in the thermodynamic limit).

Our numerical study does not allow to rigorously answer the question whether ideal amorphous Voronoi tessellations where the centroids coincide exactly with the Voronoi center might be strictly hyperuniform. Here, we list four limitations of our numerical analysis. Two of them are specific for the Quantizer problem. The other two are more general limitations:

1. A finite number of iterations of Lloyd's algorithm limits the range within which density fluctuations can cancel each other. As the system evolves under Lloyd iterations, the large-scale reorganisation that turns a hyperfluctuating into an effectively hyperuniform system is the result of an interplay between a local optimisation in each single iteration and a global trans-

port of density fluctuations. So a finite number of Lloyd iterations cannot alter the asymptotic behavior of the number variance, that is, $S(0)$. Supplementary Fig. 8 compares the structure factor at small wavenumbers for a simultaneously increasing system size and number of Lloyd iterations. Due to the exponentially fast decreasing step size of Lloyd's algorithm, we expect that the decay of the structure factor for $k \to 0$ is logarithmic (in the number of iterations and for an increasing system size). Within the first 1000 iterations the system quickly becomes effectively hyperuniform. Then, the decay of $S(k)$ for small wavenumber becomes increasingly slow. The slow convergence can be worsened by "local freezing", that is, if the step sizes of some points almost vanish due to locally optimal configurations. Such effects can partially be compensated by an adapted step size. Note also the limitations by the machine precision. A simple generalisation of Lloyd iterations using under- or overshooting is defined in references 63, 64, more complex alternatives could include, e.g., conjugate gradient descent methods.

2. Finite system sizes affect Lloyd iterations via the boundary conditions[48]. Because Lloyd iterations propagate through the entire system, the influence of the boundary cannot be avoided (e.g., by using minus sampling boundary conditions). The periodic boundary conditions minimise this effect (we have found no difference between a cubic simulation or a parallelepiped), but the remaining influence (e.g. imposed symmetries) should be assessed in further studies. In fact, due to the strong constraints of a CVT, where each Voronoi centre coincides exactly with the centre of mass, a CVT might even exhibit an extreme dependence on boundary conditions, known in the mathematics literature as *strong rigidity*. In a strongly rigid point process, the position of each point in an observation window are determined by the point configuration outside of this window (with probability one). A proof or a counter example answering the question whether CVTs are strongly rigid is beyond the scope of this analysis.

3. Finite system sizes affect also the estimates of the structure factor $S(k)$ for small wavenumbers. There are strong correlations between values of the structure factor at different k-values evaluated for the same sample. Density fluctuations at the size of the simulation box can hardly be estimated from a single sample. As stated in the Methods section, we therefore consider only wavenumbers larger than $4.5 \times k_{\text{min}}$, where k_{min} is for each simulation box the minimal value of the wavenumber. The only exceptions are the samples of the Stealthy and Determinantal PP, where we have to consider wavenumbers $k > 1.5 \times k_{\text{min}}$ because of the small system sizes.

4. The allowed wavenumbers take only discrete values, which can cause (slight) binning effects, and more importantly there are only a few allowed wave vectors with small wavenumbers. The low statistics can cause considerable statistical fluctuations, whose interpretation is non-trivial. The random variable $S(k)$ cannot be negative and is therefore obviously non-Gaussian. Therefore, the estimates of the statistical errors have to be interpreted carefully. The unbinned scattering intensity for all allowed wavenumbers are depicted in Supplementary Fig. 9. A rigorous model selection of the asymptotic behaviour is not possible with standard tools.

These limitations prevent a rigorous confirmation or negation of whether the exact CVTs in the thermodynamic limit might be strictly hyperuniform. This question remains open for further studies. Our simulations clearly demonstrate a remarkable suppression of long-range density fluctuations resulting in a system that is effectively hyperuniform.

To assess in experiments and simulations how close a system is to perfect hyperuniformity, a measure H has been established[39,78,79]. It is the ratio of $\hat{S}(0)$, a linear extrapolation of the structure factor to $k = 0$ using a least-square fit, and $S(k_{peak})$, the largest peak of the structure factor:

$$H := \frac{\hat{S}(0)}{S(k_{peak})}. \tag{14}$$

If $H \leq 10^{-3}$, the system is typically "deemed to be nearly hyperuniform", see reference 79. Note that for the reasons explained above, the linear extrapolation is chosen for simplicity and not based on a model selection, and we use only wavenumbers larger than $4.5 \times k_{\min}$ (or $k > 1.5 \times k_{\min}$ for the samples that originated from the Stealthy or Determinantal PP).

In 3D, the final configurations are, as mentioned above, almost stealthy hyperuniform. The estimated hyperuniformity index H is within statistical fluctuations consistent with $H = 0$, that is, with perfect hyperuniformity. The estimated mean values $\hat{H}$ are smaller than 10^{-4}, which is robust against variations in the ranges of the fit, e.g., using $k \in [0, 4]$ or $k \in [1.5, 45]$ (at unit number density). Our final configurations are thus among the effective hyperuniform systems with the smallest H values, see Supplementary Table 1.

In 2D, the CVTs are also almost stealthy hyperuniform. A detailed analysis at small wavenumbers with high statistics reveals a slight (but statistically significant) non-monotonic behaviour for the CVTs after a finite number of iterations of Lloyd's algorithm. It could be caused by an (almost) freezing of the density fluctuations if the local crystallites form too quickly. The stability of cell shapes close to a regular hexagon would then dramatically slow down the convergence of Lloyd iterations and thus the cancellation of the density fluctuations at large length-scales.

If we neglect the structure factor for $k < 1.5$, which is possibly strongly influenced by this effect, and extrapolate $S(k)$ to $k \to 0$ by a least-square fit of a line to the approximately linear regime $k \in [1.50, 4.50]$ (at unit number density), the estimates of H are smaller than 10^{-4}. Even the ratio of the structure factor $S(k)$ at $k = 4.5 \times k_{\min}$ (which is the smallest k vector that we use to avoid strong finite size effects), to the peak value of $S(k)$ is still smaller than 10^{-3}. The 2D CVTs quickly become (after a finite number of iterations of Lloyd's algorithm) remarkably close to hyperuniform and can be well considered as an effectively hyperuniform system.

Note that an unsaturated jammed packing of disks cannot be hyperuniform. A strictly posi-

tive probability of a defect (a missing disk), where the defects are stochastically independent from each other, destroys the hyperuniformity of the system (even if the disks are arranged on a perfect lattice). In particular, the polycrystal formed by the Lubachevsky-Stillinger packing of hard discs in Supplementary Fig. 7 is distinctly non-hyperuniform, quite in contrast to the effectively hyperuniform 2D CVT.

The effective hyperuniformity of our final tessellations is consistent with results in computer graphics in 2D, showing that Lloyd iterations can lead to structures with *blue noise*[80].

It is important, however, to note that in general the hyperuniformity condition is distinctly stronger than blue noise. The latter only requires a monotonically increasing structure factor at small wave vectors, not a vanishing of the structure factor for $k \to 0$. In particular, the standard example of blue noise, Poisson discs sampling (i.e., RSA SphP), is not hyperuniform[42]. In contrast to this, hyperuniform systems reveal a qualitatively different behaviour on large length scales.

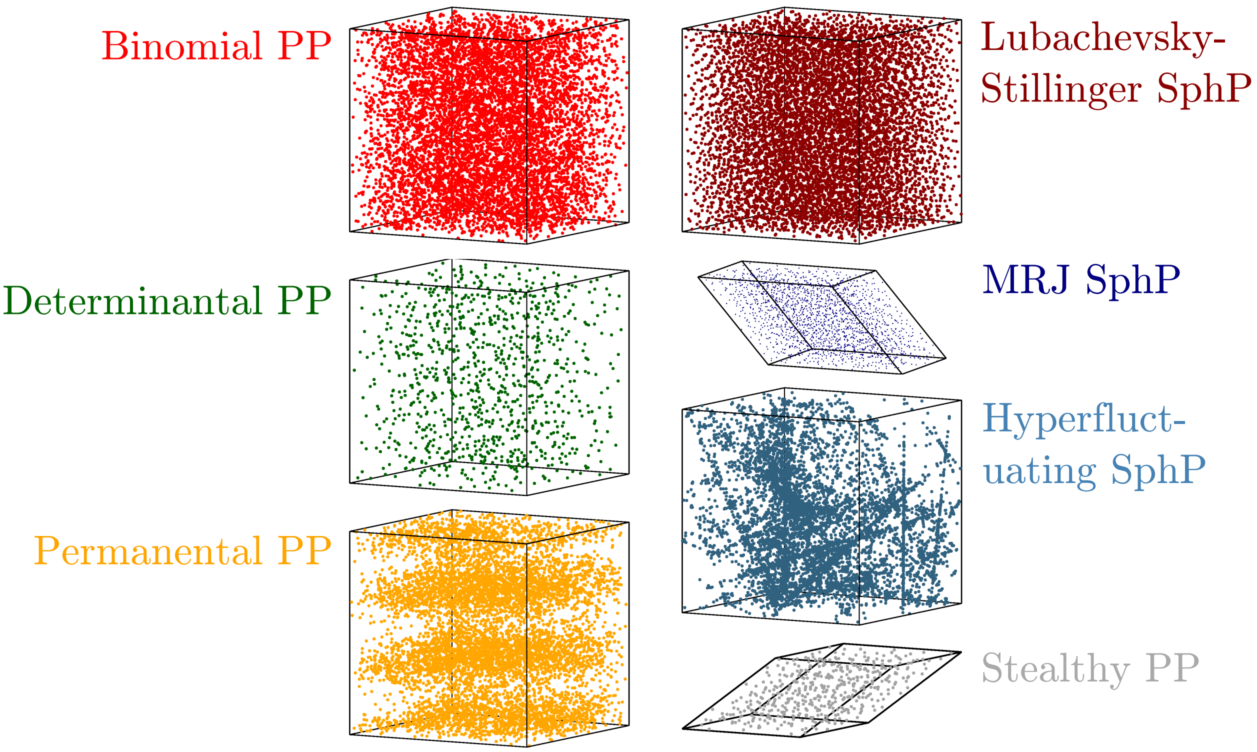

Supplementary Fig. 1 Samples of 3D initial configurations. The examples visualize the random noise in a Binomial PP, the relative homogeneity of the hard-sphere packings, as well as the anisotropy of the Permanental PP and the extreme density fluctuations in the Hyperfluctuating PP.

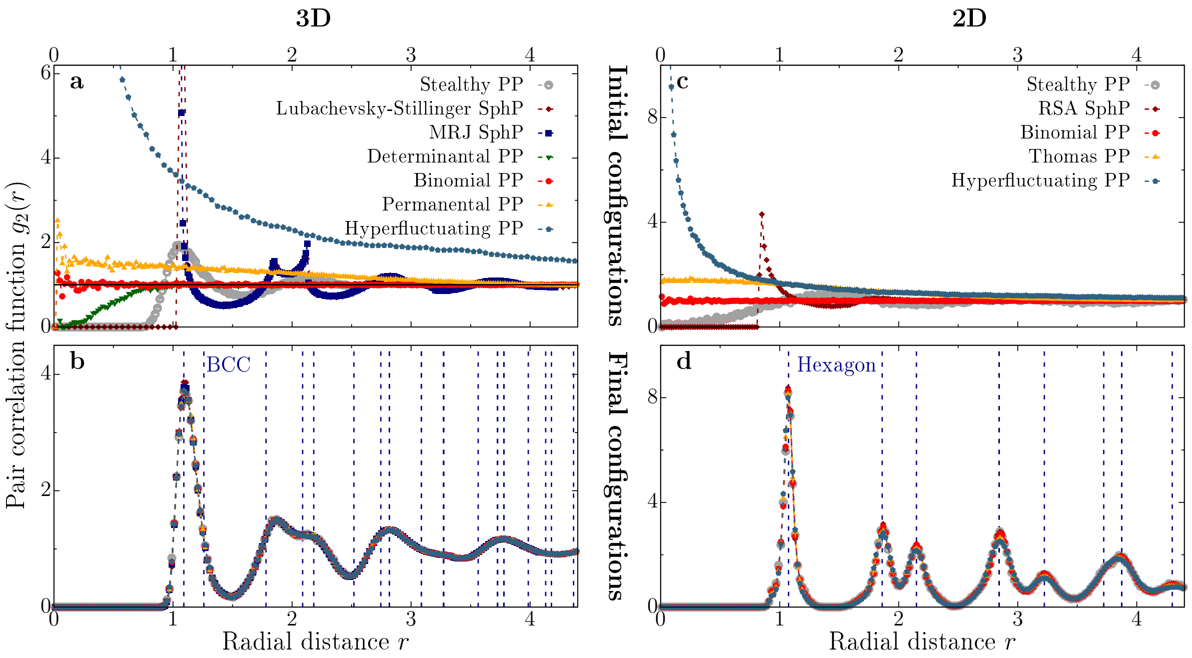

Supplementary Fig. 2 The pair-correlation function confirming the amorphousness and effective universality of the final configurations. The pair-correlation function, the real-space counterpart of the structure factor, see Figs. 3a,b and Supplementary Fig. 4, shows the effective collapse of the two-point characteristics in 3D (left) and 2D (right).

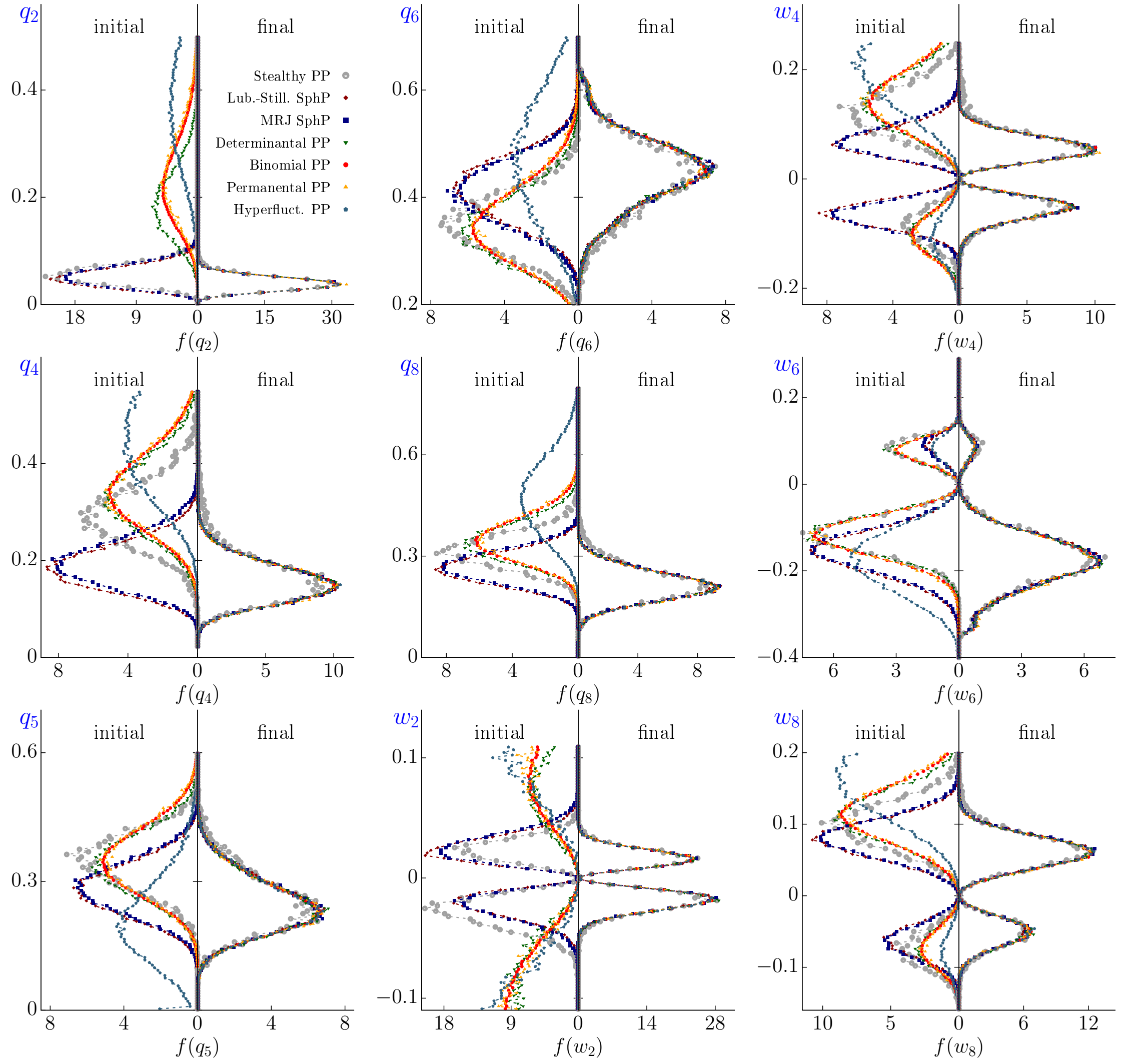

Supplementary Fig. 3 Distribution of Minkowski structure metrics supporting the amorphousness and universality of the converged states of Lloyd iterations in 3D. Shown are the rotational invariants q_l, w_l of the surface-normal Minkowski tensors which serve as structure metrics characterising the shape of the Voronoi cells. While the initial states are clearly different, the converged states are characterised by a universal smooth curve. Sharp peaks are absent which would signify the overexpression of specific structural motifs, for example, BCC ordered domains or other partial crystallinity. See Section 5 for an explanation of these metrics. Data sets and parameters as in Fig. 3.

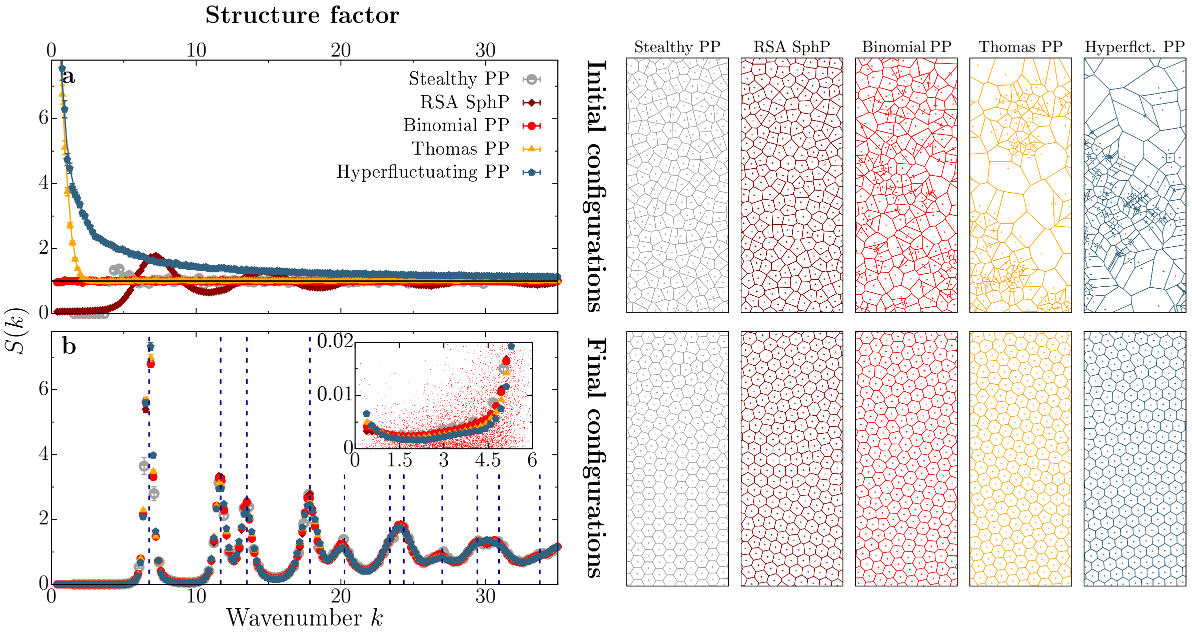

Supplementary Fig. 4 Structure factor of the 2D systems demonstrating the convergence to effectively the same universal amorphous final states that, in contrast to 3D, exhibits small-scale crystallites. The final states are statistically isotropic and homogeneous. The structure factor exhibits no Bragg peaks and converges quickly to unity for large k. The error bars represent the standard error of the mean.

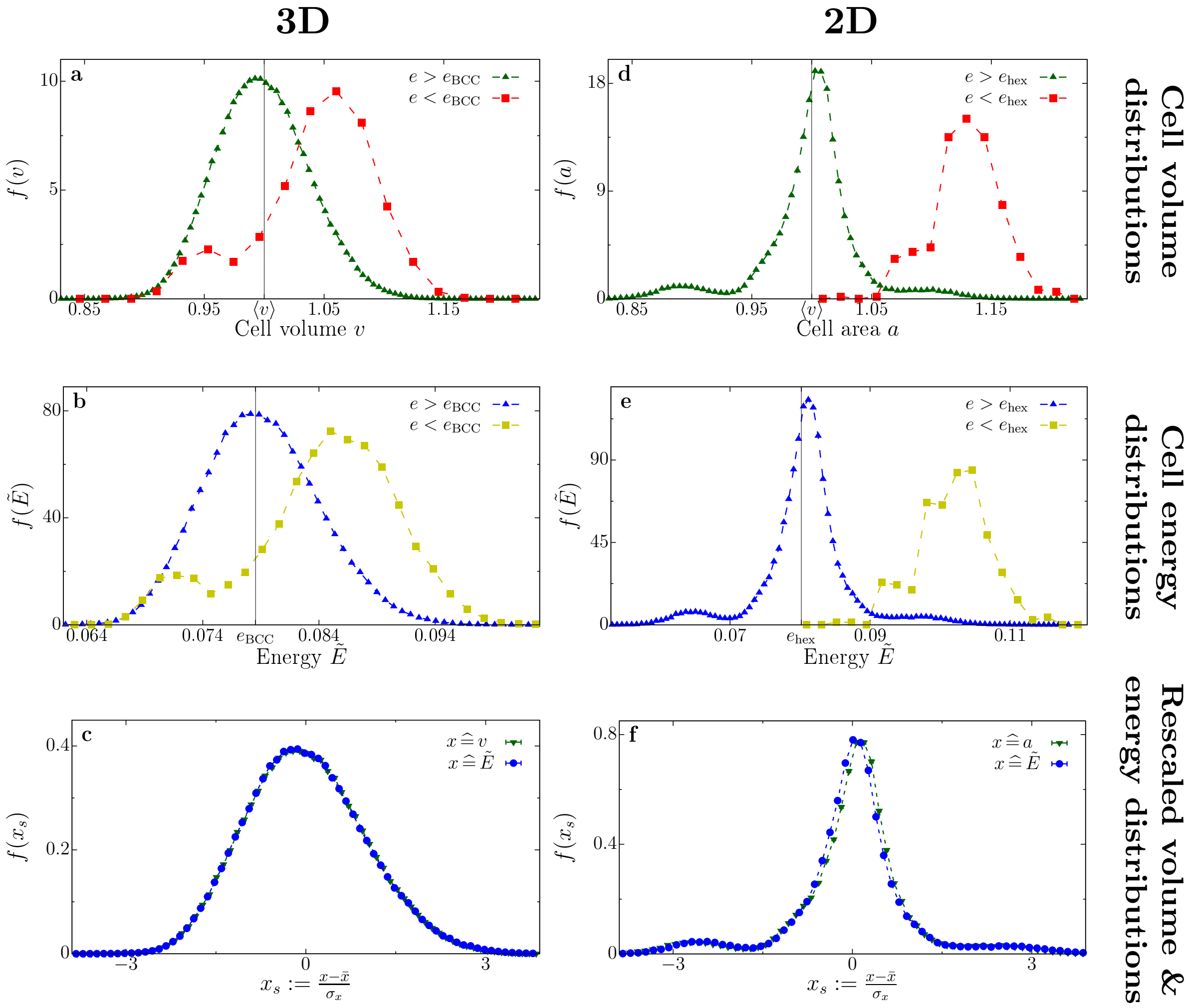

Supplementary Fig. 5 Cell energy and cell volume distributions for the final configurations as evidence for the presence of local configurations with energies below the globally optimal BCC structure. The frequency distributions f of the cell volumes v or areas a and dimensionless energies $\tilde{E}$, see equation (3), for cells with energy densities e, see equation (4), either below or above the global optimum $e_{\rm BCC}$ or $e_{\rm hex}$ in 3D or 2D, respectively. The standardized distributions of energy and the cell size **c**, **f** are indistinguishable in 3D, but in 2D there are slight but distinct differences.

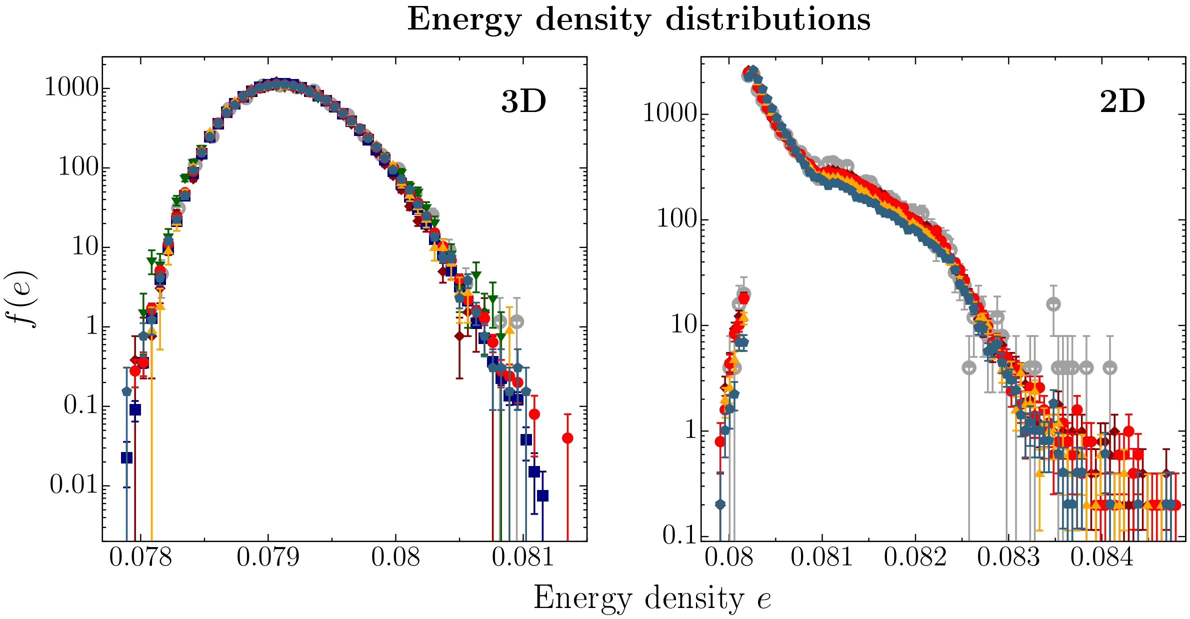

Supplementary Fig. 6 Logarithmic plot of cell energy density distributions for the final configurations in 3D and 2D. Apparently, the probability density functions have exponential tails. In 3D, all curves collapse within error bars, which represent the standard error of the mean. The empirical distribution is similar to—but distinctly different from—a gamma distribution with extreme shape parameters $O(10^5)$ and $O(10^7)$. In 2D, the distributions effectively collapse as well, but slight deviations appear to "freeze" due to the local crystallites, as explained in Sec. 7.

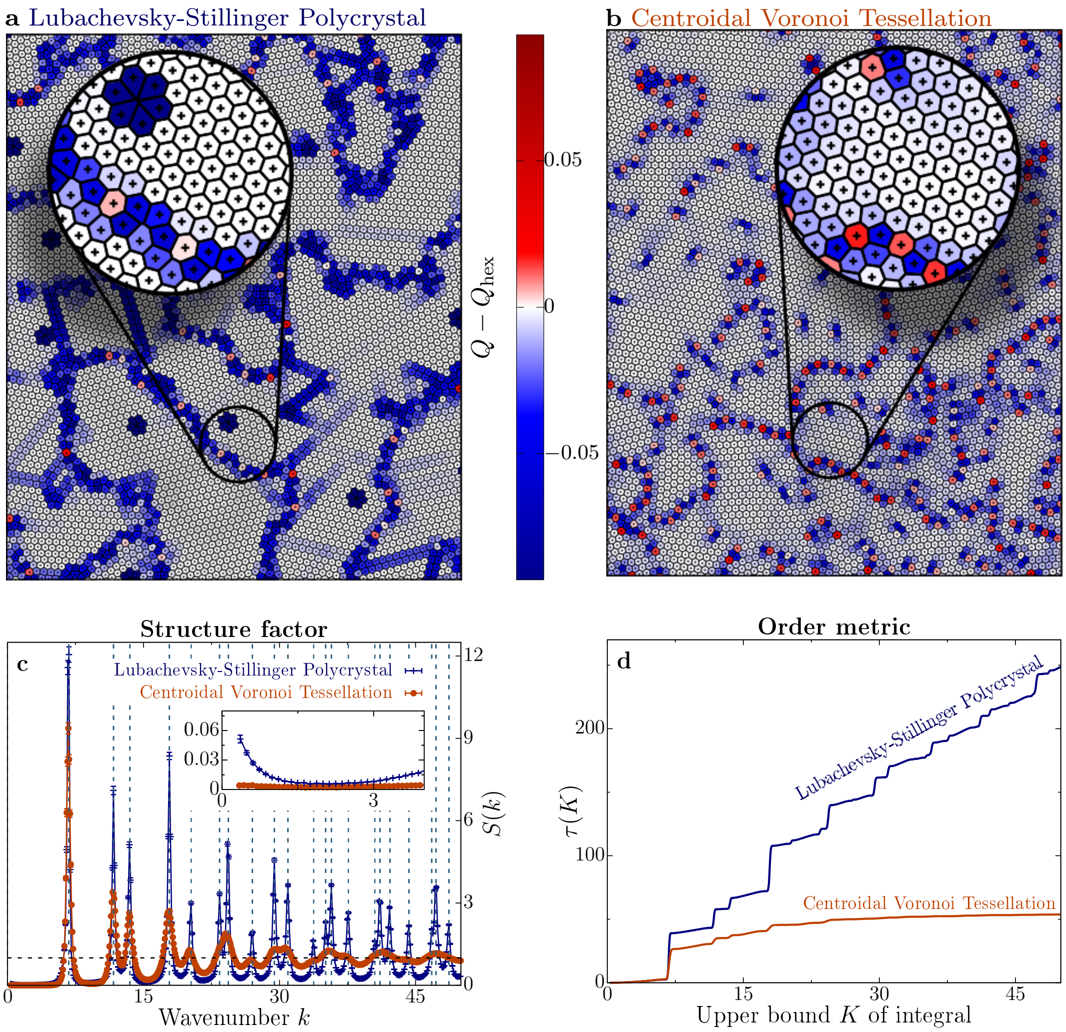

Supplementary Fig. 7 Comparison of the final configurations of Lloyd iterations to a hard-disk polycrystal. The samples of Lubachevsky-Stillinger (LS) polycrystal **a** and a Centroidal Voronoi tessellation (CVT) **b** visualize structural differences between these systems, e.g., in the domain boundaries. In the CVT, there are more cells coloured red, that is, with a better isoperimetric ration than the regular hexagon. Moreover, there are no point defects ("missing disks") in the CVT, in contrast to the LS polycrystal, appearing as dark-blue "flowers" in the Voronoi diagram **a**. The structure factor **c** exhibits distinct peaks for both systems, but they are slightly stronger and decay slower for the LS polycrystal. The order metric $\tau(K)$ as a function of the upper limit of integration K, see equation (6), does not converge for the LS polycrystal **d** because of the fixed nearest-neighbour distance. The structure factor of the CVT decays fast enough so that τ converges, revealing a more amorphous tessellation than in the hard-disk polycrystal. The error bars represent the standard error of the mean.

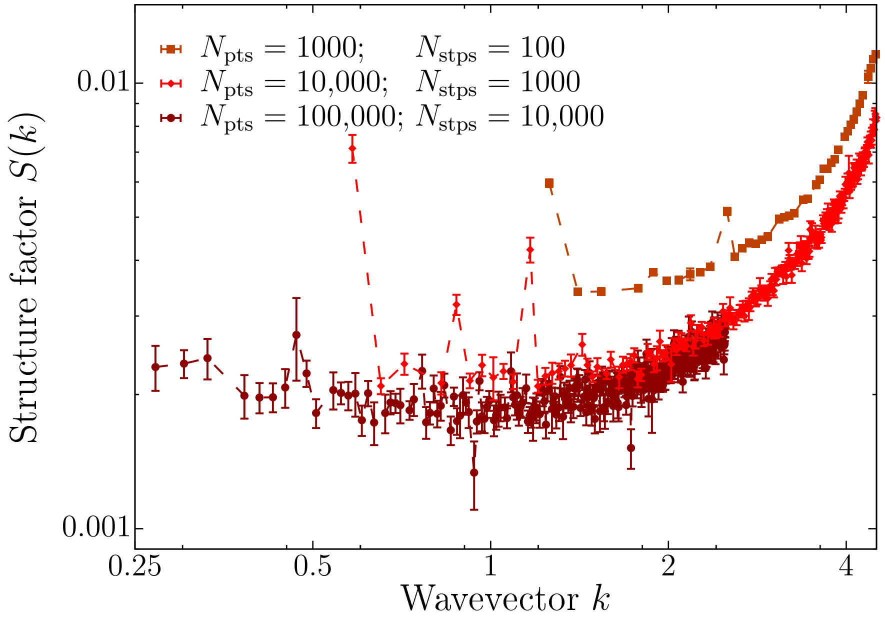

Supplementary Fig. 8 Structure factor for a simultaneously increasing number of points N_{pts} per sample and number of Lloyd iterations N_{stps}. The plot depicts for each possible wavenumber k the average structure factor $S(k)$, where the number of samples is 10^3 for $N_{pts} = 10^3$, 10^2 for $N_{pts} = 10^4$, and 30 for $N_{pts} = 10^5$. The initial configurations were realizations of a Binomial PP. A rigorous scaling is difficult due to the slow convergence of Lloyd's algorithm in the limit $k \to 0$, which can be expected due to the exponentially decaying step sizes. Intuitively speaking, for large scale density fluctuations, the larger the scale the faster increases the number of necessary steps that can compensate this fluctuation. The error bars represent the standard error of the mean.

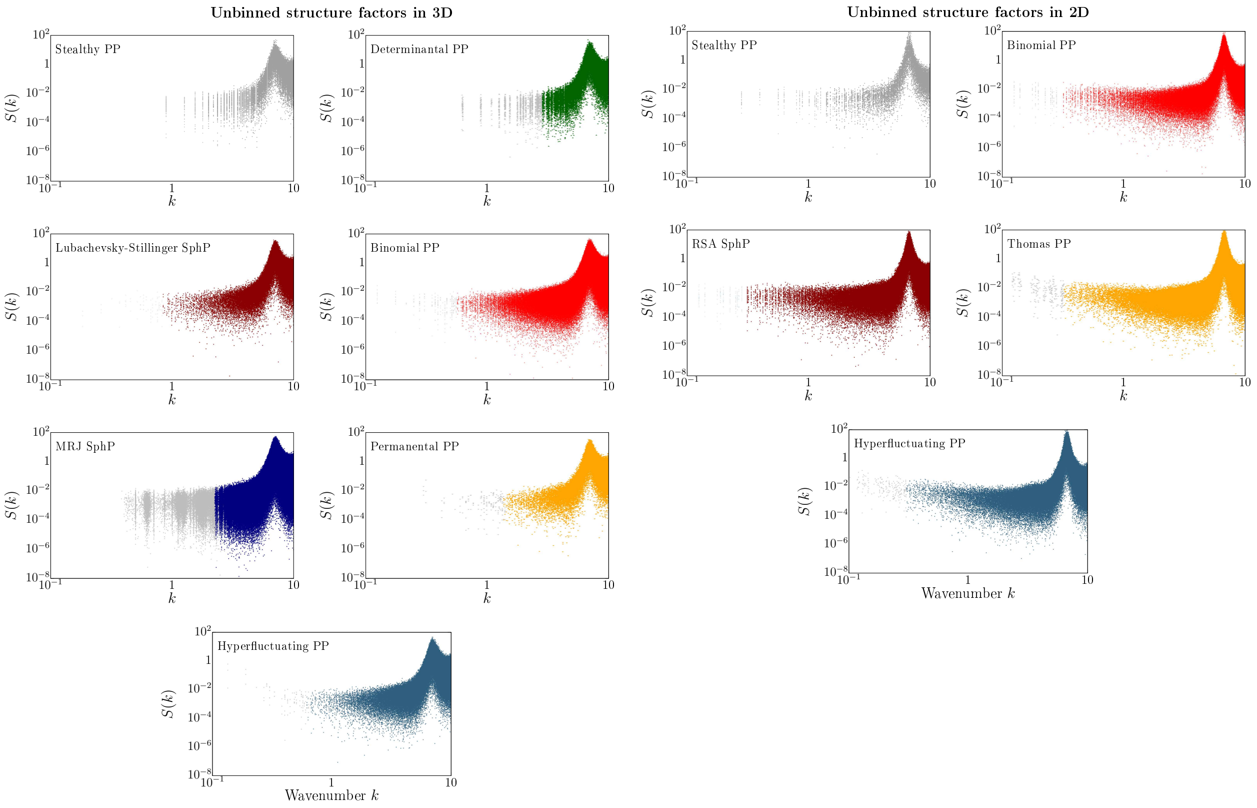

Supplementary Fig. 9 Unbinned scattering intensity of the 3D and 2D systems. Under Lloyd iterations both systems quickly become effectively hyperuniform with $S(k) < 10^{-2}$ for $k < 4$ (i.e. the structure factor at small wavenumbers drops by orders of magnitude). The plots show–for all samples of all initial configurations–the unbinned scattering intensity for all allowed wavenumbers. At the smallest wavenumbers $k < 1$, the scattering intensity is strongly affected by the limitations listed in Sec. 8, especially in 2D, where the convergence is considerably slowed down by cells that are almost hexagonal. A rigorous hypothesis test that confirms or rejects hyperuniformity is beyond the scope of this study. Gray points are used at small wavenumbers, where a strong influence of the finite system size can be expected as discussed in Sec. 8. As noted in the main text, effectively hyperuniform systems are essentially hyperuniform for all practical intents and physically relevant purposes, and are of interest in their own right.

Amorphous systems	H index
CVT	$\lesssim 10^{-4}$
Packings approaching MRJ[78]	$\lesssim 10^{-4}$
Polymer-grafted nanoparticles[81]	2×10^{-4}–10^{-2}
Polymer melt[82]	10^{-3}
Amorphous ice[79]	2×10^{-3}–4×10^{-3}
Amorphous silicon[83]	4×10^{-3}–10^{-2}
Typical liquid[84]	$\gtrsim 0.02$
RSA SphP[42]	0.05581(5)

Supplementary Table 1 ***H*** **index for effectively hyperuniform (top) and non-hyperuniform disordered systems (bottom)** We compare the H index from Eq. (10) in the Methods section for a variety of amorphous systems with suppressed density fluctuations (as mentioned in the introduction). Already after 10^4 number of Lloyd iterations, our system is among the effective hyperuniform systems with the smallest H values. In particular, this is at least two orders of magnitude smaller than for a typical liquid. The values for the H index in 2D are comparable.

3D			2D		
Initial configurations	Initial τ	Final τ	Initial configurations	Initial τ	Final τ
Stealthy PP	5.8(2)	30(1)	Stealthy PP	1.14(3)	43(4)
Lub.-Still. SphP	26.4(4)	32.3(5)			
MRJ SphP	23.7(1)	31.4(1)	RSA SphP	3.31(2)	40.4(5)
Determinantal PP	0.31(1)	31.3(7)			
Binomial PP	0	31.6(2)	Binomial PP	0	39.4(5)
Permanental PP	30(20)	31.2(8)	Thomas PP	2.9(1)	39.1(5)
Hyperfluctuating PP	∞	31.2(3)	Hyperfluctuating PP	∞	39.4(5)

Supplementary Table 2 τ **Order metric of the different initial and final configurations in 3D and 2D.** The scalar index quantifies the degree of correlations in the system both on short and large length scales. For the initial configurations, τ varies over several orders of magnitude and includes both vanishing and infinite values. The order metric converges under Lloyd iterations to the same value (≈ 31 in 3D and ≈ 40 in 2D). For the numerical integration, we chose $k \leq 16.5$ as the cut-off for the integral to avoid strong statistical fluctuations. Note that the convergence of the MRJ SphP reflects the structural features below the finite threshold. Because of the δ-peak in the pair-correlation function, τ diverges if the integration is carried out from 0 to ∞. Further systematic errors appear due to the binning. For the initial Permanental PP, strong fluctuations appear because of the strong anisotropy.

Supplementary References

1. Chiu, S., Stoyan, D., Kendall, W. & Mecke, J. *Stochastic Geometry and Its Applications*. Wiley Series in Probability and Statistics (Wiley, 2013).

2. Baddeley, A. Spatial Point Processes and their Applications. In Weil, W. (ed.) *Stochastic Geometry*, vol. 1892 of *Lecture Notes in Mathematics*, 1–75 (Springer, Berlin, 2007).

3. Illian, J., Penttinen, A., Stoyan, H. & Stoyan, D. *Statistical Analysis and Modelling of Spatial Point Patterns* (John Wiley & Sons, Chichester, 2008).

4. Last, G. & Penrose, M. *Lectures on the Poisson Process*. Institute of Mathematical Statistics Textbooks (Cambridge University Press, Cambridge, 2017).

5. Kallenberg, O. *Random Measures, Theory and Applications*, vol. 77 of *Probability Theory and Stochastic Modelling* (Springer, Cham, 2017).

6. Diggle, P. J. *Statistical Analysis of Spatial Point Patterns* (Arnold, London, 2003), 2nd edn.

7. Baddeley, A. & Vedel Jensen, E. B. *Stereology for statisticians*, vol. 103 of *Monographs on Statistics and Applied Probability* (Chapman & Hall/CRC, Boca Raton, 2004).

8. Baddeley, A., Rubak, E. & Turner, R. *Spatial point patterns: methodology and applications with R* (CRC Press, Taylor & Francis Group, Boca Raton, 2016).

9. Matsumoto, M. & Nishimura, T. Mersenne twister: A 623-dimensionally equidistributed uniform pseudo-random number generator. *ACM Trans. Model. Comput. Simul.* **8**, 3–30 (1998).

10. van der Walt, S., Colbert, S. C. & Varoquaux, G. The NumPy Array: A Structure for Efficient Numerical Computation. *Comput. Sci. Eng.* **13**, 22–30 (2011).

11. R Development Core Team. *R: A Language and Environment for Statistical Computing*. R Foundation for Statistical Computing, Vienna, Austria (2008).

12. Galassi, M. *et al.* *GNU Scientific Library: Reference Manual* (Network Theory Ltd., 2009), 3rd edn. OCLC: 552301619.

13. Boost. Boost C++ Libraries. www.boost.org (2017). Last accessed 2017-08-22.

14. Torquato, S., Zhang, G. & Stillinger, F. H. Ensemble theory for stealthy hyperuniform disordered ground states. *Phys. Rev. X* **5**, 021020 (2015).

15. Uche, O. U., Stillinger, F. H. & Torquato, S. Constraints on collective density variables: Two dimensions. *Phys. Rev. E* **70**, 046122 (2004).

16. Zhang, G., Stillinger, F. & Torquato, S. Ground states of stealthy hyperuniform potentials. i. entropically favored configurations. *Phys. Rev. E* **92**, 022119 (2015).

17. Lubachevsky, B. D. & Stillinger, F. H. Geometric properties of random disk packings. *J. Stat. Phys.* **60**, 561–583 (1990).

18. Kapfer, S. C., Mickel, W., Mecke, K. & Schröder-Turk, G. E. Jammed spheres: Minkowski tensors reveal onset of local crystallinity. *Phys. Rev. E* **85**, 030301 (2012).

19. Skoge, M., Donev, A., Stillinger, F. H. & Torquato, S. Packing hyperspheres in high-dimensional Euclidean spaces. *Phys. Rev. E* **74**, 041127 (2006).

20. Torquato, S., Truskett, T. M. & Debenedetti, P. G. Is Random Close Packing of Spheres Well Defined? *Phys. Rev. Lett.* **84**, 2064–2067 (2000).

21. Torquato, S. & Stillinger, F. H. Jammed hard-particle packings: From Kepler to Bernal and beyond. *Rev. Mod. Phys.* **82**, 2633–2672 (2010).

22. Donev, A., Stillinger, F. H. & Torquato, S. Unexpected density fluctuations in jammed disordered sphere packings. *Phys. Rev. Lett.* **95**, 090604 (2005).

23. Klatt, M. A. & Torquato, S. Characterization of maximally random jammed sphere packings: Voronoi correlation functions. *Phys. Rev. E* **90**, 052120 (2014).

24. Klatt, M. A. & Torquato, S. Characterization of maximally random jammed sphere packings. II. Correlation functions and density fluctuations. *Phys. Rev. E* **94**, 022152 (2016).

25. Torquato, S. & Jiao, Y. Robust algorithm to generate a diverse class of dense disordered and ordered sphere packings via linear programming. *Phys. Rev. E* **82**, 061302 (2010).

26. Atkinson, S., Stillinger, F. H. & Torquato, S. Detailed characterization of rattlers in exactly isostatic, strictly jammed sphere packings. *Phys. Rev. E* **88**, 062208 (2013).

27. Macchi, O. The coincidence approach to stochastic point processes. *Adv. Appl. Probab.* **7**, 83–122 (1975).

28. Dean, D. S., Doussal, P. L., Majumdar, S. N. & Schehr, G. Universal ground-state properties of free fermions in a d-dimensional trap. *Europhys. Lett.* **112**, 60001 (2015).

29. Deng, N., Zhou, W. & Haenggi, M. The Ginibre Point Process as a Model for Wireless Networks With Repulsion. *IEEE Trans. Wirel. Commun.* **14**, 107 (2015).

30. Shirai, T. & Takahashi, Y. Random point fields associated with certain Fredholm determinants I: fermion, Poisson and boson point processes. *J. Funct. Anal.* **205**, 414–463 (2003).

31. Hough, J. B., Krishnapur, M., Peres, Y. & Virág, B. Determinantal processes and independence. *Probab. Surv.* **3**, 206–229 (2006).

32. Lavancier, F., Møller, J. & Rubak, E. Determinantal point process models and statistical inference. *J. Roy. Stat. Soc. B* **77**, 853–877 (2015).

33. Scardicchio, A., Zachary, C. E. & Torquato, S. Statistical properties of determinantal point processes in high-dimensional Euclidean spaces. *Phys. Rev. E* **79**, 041108 (2009).

34. Baddeley, A. & Turner, R. spatstat: An R Package for Analyzing Spatial Point Patterns. *J. Stat. Softw.* **12**, 1 (2005).

35. McCullagh, P. and Møller, J. The permanental process. *Adv. Appl. Prob.* **38**, 873–888 (2006).

36. Klatt, M. A. *et al.* Cell Shape Analysis of Random Tessellations Based on Minkowski Tensors. In Vedel Jensen, E. B. & Kiderlen, M. (eds.) *Tensor Valuations and Their Applications in Stochastic Geometry and Imaging*, vol. 2177 of *Lecture Notes in Mathematics*, 385–421 (Springer International Publishing, Cham, 2017).

37. Schneider, R. & Weil, W. *Stochastic and Integral Geometry (Probability and Its Applications)* (Springer, Berlin, 2008).

38. Heinrich, L. & Muche, L. Second-order properties of the point process of nodes in a stationary Voronoi tessellation. *Math. Nachr.* **281**, 350–375 (2008).

39. Torquato, S. Hyperuniform states of matter. *Phys. Rep.* **745**, 1–95 (2018).

40. Heinrich, L., Schmidt, H. & Schmidt, V. Central limit theorems for Poisson hyperplane tessellations. *Ann. Appl. Probab.* **16**, 919–950 (2006).

41. Talbot, J., Tarjus, G., Van Tassel, P. R. & Viot, P. From car parking to protein adsorption: an overview of sequential adsorption processes. *Colloids Surf. A* **165**, 287–324 (2000).

42. Zhang, G. & Torquato, S. Precise algorithm to generate random sequential addition of hard hyperspheres at saturation. *Phys. Rev. E* **88**, 053312 (2013).

43. Stoyan, D. & Schlather, M. Random sequential adsorption: Relationship to dead leaves and characterization of variability. *J. Stat. Phys.* **100**, 969–979 (2000).

44. Møller, J. & Waagepetersen, R. P. *Statistical Inference and Simulation for Spatial Point Processes* (Chapman & Hall/CRC, Boca Raton, 2004).

45. Newman, D. J. The Hexagon Theorem. *IEEE Trans. Inf. Theory* **28**, 137 (1982).

46. Gersho, A. Asymptotically optimal block quantization. *IEEE Trans. Inf. Theory* **25**, 373–380 (1979).

47. Barnes, E. S. & Sloane, N. J. A. The optimal lattice quantizer in three dimensions. *SIAM J. Algebra. Discr.* **4**, 30–41 (1983).

48. Du, Q. & Wang, D. The optimal centroidal Voronoi tessellations and the Gersho's conjecture in the three-dimensional space. *Comput. Math. Appl.* **49**, 1355–1373 (2005).

49. Schröder-Turk, G. E. *et al.* Minkowski Tensor Shape Analysis of Cellular, Granular and Porous Structures. *Adv. Mater.* **23**, 2535–2553 (2011).

50. Klatt, M. A., Schröder-Turk, G. E. & Mecke, K. Mean-intercept anisotropy analysis of porous media. II. Conceptual shortcomings of the MIL tensor definition and Minkowski tensors as an alternative. *Med. Phys.* **44**, 3663–3675 (2017).

51. Jensen, E. B. V. & Kiderlen, M. (eds.) *Tensor Valuations and Their Applications in Stochastic Geometry and Imaging*, vol. 2177 of *Lecture Notes in Mathematics* (Springer International Publishing, Cham, 2017).

52. Schneider, R. *Convex Bodies: The Brunn-Minkowski Theory* (Cambridge University Press, Cambridge, 2014), 2nd edn.

53. Mickel, W., Kapfer, S. C., Schröder-Turk, G. E. & Mecke, K. Shortcomings of the bond orientational order parameters for the analysis of disordered particulate matter. *J. Chem. Phys.* **138**, 044501 (2013).

54. Torquato, S. Reformulation of the covering and quantizer problems as ground states of interacting particles. *Phys. Rev. E* **82**, 056109 (2010).

55. Gersho, A. & Gray, R. M. *Vector Quantization and Signal Compression* (Kluwer Academic Publishers, Norwell, MA, USA, 1991).

56. Pagés, G. A space quantization method for numerical integration. *J. Comput. Appl. Math.* **89**, 1–38 (1998).

57. Bollobás, B. & Stern, N. The optimal structure of market areas. *J. Econ. Theory* **4**, 174–179 (1972).

58. Honda, H. Description of cellular patterns by Dirichlet domains: The two-dimensional case. *J. Theor. Biol.* **72**, 523–543 (1978).

59. Barlow, G. W. Hexagonal territories. *Anim. Behav.* **22, Part 4**, 876 – IN1 (1974).

60. Suzuki, A. & Iri, M. Approximation of a tessellation of the plane by a Voronoi diagram. *J. Oper. Res. Soc. Japan* **29**, 69–97 (1986).

61. Du, Q., Faber, V. & Gunzburger, M. Centroidal Voronoi Tessellations: Applications and Algorithms. *SIAM Rev.* **41**, 637–676 (1999).

62. Hasegawa, M. & Tanemura, M. On the pattern of space division by territories. *Ann. Inst. Statist. Math.* **28**, 509–519 (1976).

63. Tanemura, M. & Hasegawa, M. Geometrical models of territory I. Models for synchronous and asynchronous settlement of territories. *J. Theor. Biol.* **82**, 477–496 (1980).

64. Okabe, A., Boots, B., Sugihara, K. & Chiu, S. N. *Spatial Tessellations: Concepts and Applications of Voronoi Diagrams* (Wiley, Chichester; New York, 2000), 2nd edn.

65. Sigmund, O. & Hougaard, K. Geometric Properties of Optimal Photonic Crystals. *Phys. Rev. Lett.* **100**, 153904 (2008).

66. Graf, S. & Luschgy, H. *Foundations of quantization for probability distributions*. No. 1730 in Lecture notes in mathematics (Springer, Berlin; New York, 2000).

67. Barber, C. B., Dobkin, D. P. & Huhdanpaa, H. T. The Quickhull algorithm for convex hulls. *ACM Trans. on Mathematical Software* **22**, 469–483 (1996). http://www.qhull.org.

68. Rycroft, C. H., Grest, G. S., Landry, J. W. & Bazant, M. Z. Analysis of granular flow in a pebble-bed nuclear reactor. *Phys. Rev. E* **74**, 021306 (2006).

69. Rycroft, C. H. VORO++: A three-dimensional Voronoi cell library in C++. *Chaos* **19** (2009).

70. MATLAB and Statistics Toolbox Release 2017b, The MathWorks, Inc., Natick, Massachusetts, United States.

71. Sommer, H. J. polygeom.m. *MATLAB Central File Exchange* (2016). Retrieved July 10, 2017 from http://www.mathworks.com/matlabcentral/fileexchange/319.

72. Becker, A. T. lloydsalgorithm. *MATLAB Central File Exchange* (2015). Retrieved July 31, 2017 from http://www.mathworks.com/matlabcentral/fileexchange/41507.

73. Schröder-Turk, G. E., Kapfer, S., Breidenbach, B., Beisbart, C. & Mecke, K. Tensorial minkowski functionals and anisotropy measures for planar patterns. *J. Microsc.* **238**, 57–74 (2010).

74. Schröder-Turk, G. E. *et al.* Minkowski tensors of anisotropic spatial structure. *New J. Phys.* **15**, 083028 (2013).

75. Mickel, W. *et al.* Robust Pore Size Analysis of Filamentous Networks from Three-Dimensional Confocal Microscopy. *Biophys. J.* **95**, 6072–6080 (2008).

76. Schaller, F. M. *et al.* Set Voronoi diagrams of 3d assemblies of aspherical particles. *Philos. Mag.* **93**, 3993–4017 (2013). http://theorie1.physik.fau.de/research/pomelo/index.html.

77. Zhang, J., Emelianenko, M. & Du, Q. Periodic Centroidal Voronoi Tessellations. *Int. J. Num. Analysis Modeling* **9**, 950 (2012).

78. Atkinson, S., Zhang, G., Hopkins, A. B. & Torquato, S. Critical slowing down and hyperuniformity on approach to jamming. *Phys. Rev. E* **94**, 012902 (2016).

79. Martelli, F., Torquato, S., Giovambattista, N. & Car, R. Large-scale structure and hyperuniformity of amorphous ices. *Phys. Rev. Lett.* **119**, 136002 (2017).

80. Hiller, S., Deussen, O. & Keller, A. Tiled Blue Noise Samples. In *Proceedings of the Vision Modeling and Visualization Conference 2001*, VMV '01, 265–272 (Aka GmbH, 2001).

81. Chremos, A. & Douglas, J. F. Particle localization and hyperuniformity of polymer-grafted nanoparticle materials: Particle localization and hyperuniformity of polymer-grafted nanoparticle materials. *Ann. Phys.* **529**, 1600342 (2017).

82. Xu, W.-S., Douglas, J. F. & Freed, K. F. Influence of Cohesive Energy on the Thermodynamic Properties of a Model Glass-Forming Polymer Melt. *Macromolecules* **49**, 8341–8354 (2016).

83. Xie, R. *et al.* Hyperuniformity in amorphous silicon based on the measurement of the infinite-wavelength limit of the structure factor. *Proc. Natl. Acad. Sci. USA* **110**, 13250–13254 (2013).

84. Hansen, J. & McDonald, I. *Theory of Simple Liquids* (Elsevier Science, 2006).
