# Supplementary material for: Universal hidden order in amorphous cellular geometries
Source: Nat Commun. 2019 Feb 18;10:811. doi: 10.1038/s41467-019-08360-5 (PMC6379405; doi:10.1038/s41467-019-08360-5)
Supplement: Supplementary file 3 — Description of Additional Supplementary Files [file 41467_2019_8360_MOESM3_ESM.pdf]

Description of Additional Supplementary Files

Supplementary Movie 1

Lloyd’s iterations quickly convert a hyperfluctuating tessellation into an effectively hyperuni-form structure.

The video combines the intuitive geometrical picture with a rigorous mathematical analysis. It visualizes how an iterative optimisation of the single cells leads to a global reorganisa-tion. The global behaviour is reverted, density fluctuations cancel each other, and a hidden global order is formed. The hyperfluctuating system becomes disordered effectively hyper-uniform. An initial cluster of Voronoi cells is visualized in the left panel (rotating until 00:16). The structure factor of the underlying hyperfluctuating point process diverges for a vanish-ing wavenumber k (right bottom panel). The tessellation exhibits strong volume fluctuations (right top panel) and a high Quantizer energy (green dot in bottom panel) compared to the Quantizer energy of the BCC lattice (dotted line in bottom panel). Then, Lloyd’s itera-tions iteratively optimise the cells (00:17–00:37). Thus, density fluctuations are transported through the system, the energy converges, and the volume fluctuations almost vanish. The final configuration remains amorphous (rotating 00:38–01:00), but a hidden long-range or-der evolves. The tessellation becomes effectively hyperuniform. In the second part of the video (01:00–02:07), the evolving cell configuration (bottom panels) is directly compared to the initial configuration (top panels). This highlights the surprising formation of effective hyperuniformity in a system that was initially anti-hyperuniform.
